# Mapping the interplay of atrial fibrillation, brain structure, and cognitive dysfunction

**DOI:** 10.1002/alz.13870

**Published:** 2024-06-05

**Authors:** Marvin Petersen, Céleste Chevalier, Felix L. Naegele, Thies Ingwersen, Amir Omidvarnia, Felix Hoffstaedter, Kaustubh Patil, Simon B. Eickhoff, Renate B. Schnabel, Paulus Kirchhof, Eckhard Schlemm, Bastian Cheng, Götz Thomalla, Märit Jensen

**Affiliations:** ^1^ Department of Neurology University Medical Center Hamburg‐Eppendorf Hamburg Germany; ^2^ Department of Cardiology University Heart and Vascular Center Hamburg Germany; ^3^ DZHK (German Center for Cardiovascular Research), partner site Hamburg/Kiel/Luebeck Hamburg Germany; ^4^ Institute for Systems Neuroscience, Medical Faculty Heinrich‐Heine University Düsseldorf Düsseldorf Germany; ^5^ Institute of Neuroscience and Medicine Brain and Behaviour (INM‐7), Research Center Jülich Jülich Germany

**Keywords:** atrial fibrillation, diffusion magnetic resonance imaging, neuroimaging, neuropsychological assessment, structural magnetic resonance imaging

## Abstract

**INTRODUCTION:**

Atrial fibrillation (AF) is associated with an elevated risk of cognitive impairment and dementia. Understanding the cognitive sequelae and brain structural changes associated with AF is vital for addressing ensuing health care needs.

**METHODS AND RESULTS:**

We examined 1335 stroke‐free individuals with AF and 2683 matched controls using neuropsychological assessments and multimodal neuroimaging. The analysis revealed that individuals with AF exhibited deficits in executive function, processing speed, and reasoning, accompanied by reduced cortical thickness, elevated extracellular free‐water content, and widespread white matter abnormalities, indicative of small vessel pathology. Notably, brain structural differences statistically mediated the relationship between AF and cognitive performance.

**DISCUSSION:**

Integrating a comprehensive analysis approach with extensive clinical and magnetic resonance imaging data, our study highlights small vessel pathology as a possible unifying link among AF, cognitive decline, and abnormal brain structure. These insights can inform diagnostic approaches and motivate the ongoing implementation of effective therapeutic strategies.

**Highlights**

- We investigated neuropsychological and multimodal neuroimaging data of 1335 individuals with atrial fibrillation (AF) and 2683 matched controls.
- Our analysis revealed AF‐associated deficits in cognitive domains of attention, executive function, processing speed, and reasoning.
- Cognitive deficits in the AF group were accompanied by structural brain alterations including reduced cortical thickness and gray matter volume, alongside increased extracellular free‐water content as well as widespread differences of white matter integrity.
- Structural brain changes statistically mediated the link between AF and cognitive performance, emphasizing the potential of structural imaging markers as a diagnostic tool in AF‐related cognitive decline.

## 1 BACKGROUND

The association between cardiovascular health and cognitive function has gained increasing attention. Atrial fibrillation (AF), the most common cardiac arrhythmia, affects > 37 million people worldwide, making it a substantial public health concern.^1^ Several meta‐analyses indicate that AF is associated with incidence of cognitive decline and dementia even when accounting for prevalent stroke and shared risk factors.^2^, ^3^, ^4^, ^5^ Given that treatments can alter the progression of AF, comprehending its impact on the brain is vital for effective prevention and management of cognitive sequelae.

Mechanistic models have been proposed to explain the connection between AF and disorders of cognition. The connection is considered to arise from AF promoting a prothrombotic and proinflammatory environment, reduced cardiac output, and subsequent cerebral perturbations like hypoperfusion, inflammation, blood–brain barrier leakage, endothelial dysfunction, and small vessel pathology.^6^, ^7^, ^8^

Magnetic resonance imaging (MRI) allows for mapping structural brain injury, which potentially mediates cognitive effects found in AF. Although many studies suggest a link of AF with lower global brain volume and increased small vessel disease burden, the understanding of the association is still limited.^6^, ^9^, ^10^ Existing research often includes AF individuals with concurrent ischemic stroke, potentially confounding results. Studies also largely focus on global brain structural measures, disregarding the potential importance of regionally specific changes. Last, small sample sizes in many studies may have led to inconsistent findings.

We argue that for a better understanding of the interplay among AF, cognition, and brain structure, analyses are needed that unify (1) large‐scale, population‐based cognitive and MRI data to address confounding and ensure reproducibility; (2) broad cognitive phenotyping; and (3) advanced neuroimaging techniques to comprehensively characterize the pathomechanistic correlates of AF.^11^, ^12^

Tapping into these research needs, our study aims to advance the understanding of AF‐related cognitive impairments by investigating an AF sample from the UK Biobank (UKB) in a case–control design, leveraging broad cognitive phenotyping and advanced neuroimaging markers of tissue macro‐ and microstructure on global and regional scales.

## 2 METHODS

### 2.1 Study population

We examined cross‐sectional clinical and imaging data from the UKB (age 45–80 years).^13^ To reduce confounding effects, participants with either a history or a current diagnosis of neurological or psychiatric diseases including history of stroke and dementia were excluded (Table S1 in supporting information; http://biobank.ndph.ox.ac.uk/showcase/coding.cgi?id=6). Individuals with AF were identified based on International Classification of Diseases, 10th revision (IC‐D1‐0) diagnoses. A healthy control sample was compiled performing a 1:2 propensity score matching specifically accounting for confounders known to affect cognitive performance as well as anatomical and diffusion MRI indices. Matching criteria included age, sex, education, systolic blood pressure, and smoking behavior, as well as blood cholesterol and glucose levels, using the matchit package in R (v4.3.3).^14^

### 2.2 Ethics approval

The UKB was ethically approved by the North West Multi–Centre Research Ethics Committee (MREC) and written informed consent was obtained from all participants. Details on the UKB Ethics and Governance framework are provided online (https://www.ukbiobank.ac.uk/media/0xsbmfmw/egf.pdf).^15^ Data usage is covered by a vote of the ethics committee of the medical faculty of the Heinrich Heine University Düsseldorf.

### 2.3 Cognitive assessments

The UKB uses computerized versions of established tests to evaluate multiple cognitive domains. Here we investigated cognitive scores of attention and executive function (Tower Rearranging Test, Trail Making Test Part B), processing speed (Reaction Time Test, Digit Symbol Substitution Test, Trail Making Test Part A), memory (Numeric Memory Test, Paired Associate Learning Test, Prospective Memory Test), and reasoning (Fluid Intelligence Test, Matrix Pattern Completion Test).^16^ Detailed descriptions of the individual tests can be found elsewhere.^17^ Results on the Trail Making Tests and the Reaction Time Test were inverted to ensure that high scores correspond with better cognitive performance across all tests. Within the four domains, respective tests were *z* scored and averaged to obtain domain scores as has been done previously.^18^

### 2.4 Brain imaging

The full UKB neuroimaging protocol can be found online (https://biobank.ctsu.ox.ac.uk/crystal/crystal/docs/brain_mri.pdf).^13^ In brief, 3D T1‐weighted rapid acquisition gradient‐echo sequence (MPRAGE, 1 × 1 × 1 mm, 192 × 256 × 256, repetition time = 2000 ms, echo time = 2.01 ms), and multishell diffusion weighted imaging (DWI; 2 × 2 × 2 mm, 104 × 104 × 72, repetition time = 3600 ms, echo time = 92 ms, 50 diffusion‐encoding directions—50x b = 1000 s/mm^2^ and 50x b = 2000 s/mm^2^) were acquired on a 3T Siemens Skyra MRI scanner (Siemens).

**FIGURE 1 alz13870-fig-0001:**
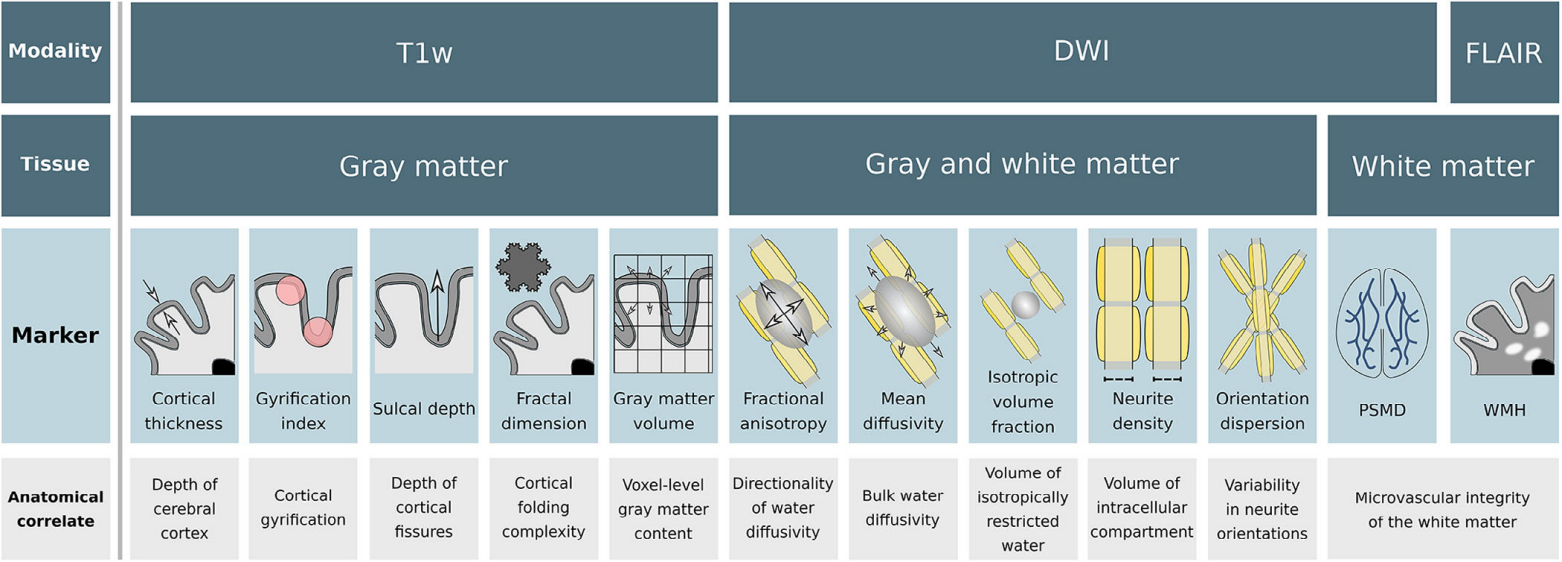
Schematic illustration of the investigated imaging markers. To assess the cerebral gray and white matter, micro‐ and macrostructural imaging markers were derived. In the first row, the schematic outlines the specific imaging sequences used to derive the imaging markers. These sequences form the basis for the analysis of the various cerebral structures. The second row delineates the tissues from which the imaging markers were extracted. The third row provides diagrammatic representations of the markers, detailing their specific definitions and functions: cortical thickness is defined as the distance between the pial surface and the boundary between white matter and gray matter; the gyrification index quantifies the folding complexity within the cerebral cortex; sulcal depth measures the depth of the brain's sulci; fractal dimension informs about the complexity and regularity of cortical folding; gray matter volume estimations are based on voxel‐based morphometry using local intensity and registration information to serve as a proxy for brain tissue composition; FA assesses the directional bias of diffusion; MD denotes the rate of molecular diffusion; the isotropic volume fraction indicates the proportion of isotropic diffusion, reflecting the content of free extracellular water within brain tissue; the neurite density index represents the proportion of water diffusion within neurites, indicative of cellularity and tissue density; orientation dispersion measures the variability in the primary directions of water diffusion, reflecting the coherence or dispersion of neurite orientations. PSMD is a diffusion metric that gauges white matter integrity by mirroring the distribution of mean diffusivity along the white matter skeleton. WMH load refers to the amount of T2‐hyperintense lesions, indicative of abnormalities in the white matter resulting from small vessel pathology. Anatomical correlates of the respective imaging markers are described in the fourth row. Modified from Petersen et al.^12^ Abbreviations: DWI, diffusion‐weighted imaging; FA, fractional anisotropy; FLAIR, fluid‐attenuated inversion recovery; MD, mean diffusivity; PSMD, peak width of skeletonized mean diffusivity; T1w, T1‐weighted imaging; WMH, white matter hyperintensity.

A summary of the imaging markers obtained for both gray and white matter is depicted in Figure 1. For an in‐depth explanation of image preprocessing, the calculation of macro‐ and microstructural indices, and quality assurance see Text S1 in supporting information.

Based on T1w images, imaging markers of cortical macrostructure were computed with the Computational Anatomy Toolbox for SPM (CAT12).^19^ Macrostructural measures inform about large‐scale anatomy and geometric characteristics of the brain. Cortical thickness (CT) was measured as the shortest difference between the pial surface and the boundary between white matter and gray matter.^20^ Three measures of cortical folding geometry were obtained: (1) the gyrification index measuring the amount of gyrification, (2) the sulcal depth measuring the depth of the brain's sulci, and (3) the fractal dimension (FD) informing about complexity and regularity of cortical folding.^21^, ^22^, ^23^ Leveraging voxel‐based morphometry (VBM), local intensity and registration information were used to obtain gray matter volume estimates as a proxy of cortical tissue volume.^24^, ^25^ For the statistical analysis, gray matter macrostructural measures were averaged across the whole brain (global level), as well as averaged within Schaefer400 atlas regions of interests (ROIs).^26^

Based on DWI, voxel‐level imaging markers of tissue microstructure were obtained, that is, markers representing the underlying organization and arrangement of cells, neurites, and other microscopic components within brain tissue. After DWI preprocessing, conventional diffusion tensor imaging (DTI) markers of white matter microstructure, that is, fractional anisotropy (FA) and mean diffusivity (MD), were derived that have been extensively used in neuroscientific and neuropsychological research.^27^, ^28^ In addition, neurite orientation dispersion and density imaging (NODDI) was used to obtain diffusion measures with higher tissue specificity.^29^ NODDI models (1) the neurite density index (also intracellular volume fraction [ICVF]) representing the proportion of water diffusion within neurites, indicative of cellularity and tissue density; (2) isotropic volume fraction (ISOVF) indicating the proportion of isotropic diffusion which quantifies the extracellular free‐water content within brain tissue; as well as (3) the orientation dispersion measuring the variability in the primary directions of water diffusion reflecting the coherence or dispersion of neurite orientations. For further statistical analysis, voxel‐level microstructural markers were averaged in cortical Schaefer400 atlas regions, as well as 70 predefined anatomical white matter tracts of the normative HCP‐842 tractography atlas.^26^, ^30^ In addition, microstructural markers were projected on a representative skeleton of the entire white matter derived by tract‐based spatial statistics (TBSS).^31^ To obtain global markers of gray matter and white matter microstructure, the measures were averaged across the whole gray matter and all voxels of the representative white matter skeleton, respectively.

Finally, the peak width of skeletonized mean diffusivity (PSMD) as well as normalized volumes of white matter hyperintensities (WMH load) were obtained as surrogate markers of microvascular injury in the white matter.^32^, ^33^, ^34^

**RESEARCH IN CONTEXT**

1. **Systematic review**: We reviewed publications from PubMed and public search engines investigating the relationships among atrial fibrillation (AF), cognitive performance, and brain structure. Previous studies suggest that AF increases the risk of cognitive decline and dementia, yet the links among AF, cognitive impairment, and structural brain changes remain to be characterized.
2. **Interpretation**: Our findings indicate an independent association between AF and cognitive deficits that is mediated by specific macro‐ and microstructural brain alterations. These insights could guide diagnostics and treatment strategies and generate new hypotheses for cross‐disciplinary research.
3. **Future directions**: This study serves as a starting point for further explorations in the field. Potential research trajectories include: (1) pinpointing surrogate biomarkers within the specified brain structures to inform clinical investigations, (2) performing correlational studies that integrate histopathological and neuroimaging data to enhance our comprehension of the neural foundations of AF, and (3) mitigating the adverse effects of AF on brain health through imaging‐informed diagnostic and therapeutic approaches.

### 2.5 Statistical analysis

All statistical[Fig alz13870-fig-0001] analyses were conducted in Python 3.9.1 as well as FSL's Permutation Analysis of Linear Models (PALM) based on MATLAB v.2021b.^35^, ^36^ Statistical tests were two‐sided, with *P *< 0.05 as significance threshold. To account for multiple comparisons *P* values were adjusted via false discovery rate correction.^37^

#### 2.5.1 Demographic data

Sample characteristics were compared between AF individuals and healthy controls using *χ^2^* tests (binary) and two‐sample *t* tests (continuous). Clinical variables were compared between groups in separate analyses of covariance (ANCOVA) adjusted for age, sex, education, and cardiovascular risk factors (systolic and diastolic blood pressure, smoking behavior, blood cholesterol, high‐density lipoprotein, low‐density lipoprotein, blood glucose).

#### 2.5.2 Imaging

Statistical analysis of imaging parameters was performed in two stages. First, global measures, that is, mean cortical macrostructural markers, mean cortical microstructural markers, mean skeletonized microstructural parameters, WMH load, and PSMD, were compared between individuals with AF and healthy controls in separate ANCOVAs, adjusted for age, sex, education, and cardiovascular risk. In the case of gray matter volume, total intracranial volume served as an additional covariate.

In a mediation analysis, we tested whether the relationship of AF and cognitive domain scores was mediated by global imaging parameters.^38^ A mediation analysis decomposes the total effect of AF on a cognitive domain performance into two components: (1) the direct, that is, non‐mediated, effect of AF on cognitive domain scores, and (2) the indirect effect, that is, the portion of the effect that can be attributed to the global imaging parameters. Thus, it allows us to disentangle the complex interplay between AF and cognitive function, enabling the examination of brain structure as a potentially relevant intermediary in this link. An indirect effect was considered to mediate the relationship between AF and cognition when AF was significantly associated to the mediator, the mediator was significantly associated to the cognitive domain, and the link between AF and the cognitive domain was reduced (partial mediation) or became non‐significant (full mediation) when controlling for the mediator. The analysis was performed post hoc, that is, only scores of cognitive domains that significantly differed between the groups were considered. The presence of a significant mediating effect was determined using bootstrapping (n_bootstrap _= 5000). Models were adjusted for age, sex, education, and cardiovascular risk.

Next, we examined spatial patterns of brain structural changes linked to AF. We performed ROI‐level permutation‐based (n_permutation _= 5000) testing for two‐sided group differences of cortical macrostructural and microstructural markers averaged in Schaefer400 atlas regions as well as white matter microstructural markers in 70 predefined anatomical white matter tracts, respectively. In addition, we performed whole‐brain voxel‐wise testing of skeletonized microstructural markers conducting TBSS.^31^ TBSS was performed based on the same design matrices as in the ROI‐level analysis, that is, performing non‐parametric (n_permutation _= 5000) two‐sided group comparisons between cases and controls, with threshold‐free cluster enhancement (TFCE) and false discovery rate correction.^39^

### 2.6 Sensitivity analysis

We conducted a sensitivity analysis to ascertain the independence of our results from AF‐related comorbidities considered to impact cognitive performance and brain health. To this end, we controlled for the diagnosis of comorbidities alongside demographics and cardiovascular risk factors in the statistical models comparing cognitive domain scores and global imaging markers. The comorbidities considered encompassed atherosclerotic heart disease, congestive heart failure, hyperthyroidism, diabetes mellitus type 2, alcohol abuse, and chronic obstructive sleep apnea.

### 2.7 Data availability

UKB data can be obtained via its standardized data access procedure (https://www.ukbiobank.ac.uk/).

## 3 RESULTS

### 3.1 Sample characteristics

After sample selection, quality assessment, and matching, the final analysis sample included 1335 individuals with AF (30% female, mean age 69.1 years) and 2683 matched controls (31% female, mean age 69.1 years; see Table 1). For a flow chart on the sample selection procedure and balance plots detailing matching results refer to Figures S1 and S2, respectively, in supporting information. After matching, groups were comparable in terms of age, sex, years of education, and cardiovascular risk factors. Information on AF‐related comorbidities is displayed in Table S2 in supporting information.

**TABLE 1:** Sample characteristics of AF individuals and matched controls.

Measure^*^	Atrial fibrillation^*^	Matched controls^*^	*P_uncorr_* ^a^	*P_FDR_* ^b^	Cohen *d or χ^2^*
Age, years	69.06 ± 6.60 (1335)	69.05 ± 6.37 (2683)	0.989	0.989	<0.01
Women, *n*, %	404, 30.26 (1335)	830, 30.94 (2683)	0.690	0.989	0.16
Education (ISCED)	4.29 ± 1.55 (1335)	4.30 ± 1.49 (2683)	0.849	0.989	0.01
Systolic blood pressure, mmHg	142.06 ± 19.11 (998)	142.83 ± 18.57 (2161)	0.288	0.721	0.04
Diastolic blood pressure, mmHg	79.57 ± 11.39 (979)	78.51 ± 10.53 (2104)	0.014	0.072	−0.10
Current smokers, *n*, %	26, 1.95 (1320)	52, 1.94 (2657)	0.920	0.989	0.01
Blood cholesterol, mmol/L	5.53 ± 1.10 (1257)	5.54 ± 1.08 (2497)	0.959	0.989	<0.01
High density lipoprotein, mmol/L	1.39 ± 0.36 (1142)	1.41 ± 0.36 (2289)	0.069	0.229	0.07
Low density lipoprotein, mmol/L	3.46 ± 0.84 (1255)	3.47 ± 0.83 (2490)	0.964	0.989	<0.01
Blood glucose, mmol/L	5.08 ± 1.09 (1143)	5.08 ± 1.06 (2287)	0.987	0.989	<0.01

Abbreviations: AF, atrial fibrillation; ISCED, International Standard Classification of Education; SD, standard deviation.

^a^ Uncorrected *P* values of t‐tests or *χ2* tests.

^b^ False discovery rate‐corrected *P* values of *t* tests or *χ^2^* tests.

* Presented as mean ± SD (*n* of datapoints).

### 3.2 Cognitive function

Individuals with AF showed worse test performance for the cognitive domains of attention/executive function (mean ± standard deviation [SD], −0.058 ± 0.851 vs. 0.039 ± 0.820, Cohen *d* = 0.12, *P_FDR_* = 0.012) and reasoning (mean ± SD, −0.085 ± 0.837 vs. 0.049 ± 0.838, Cohen *d* = 0.16, *P_FDR_* < 0.001) than matched controls (see Table 2). Domain scores of memory function and information processing speed showed no significant differences between groups. These results remained stable when additionally controlling for AF comorbidities (Table S3 in supporting information). On the level of individual cognitive tests of information processing speed, the AF group showed lower performance on the Digit Symbol Substitution Test (mean ± SD, 16.68 ± 5.08 vs. 17.28 ± 5.04, Cohen *d* = 0.12, *P_FDR_* = 0.034). More details on individual cognitive test performance can be found in Table S4 in supporting information.

**TABLE 2:** Results of cognitive and imaging assessments of individuals with atrial fibrillation compared to matched controls.

Clinical measure	Atrial fibrillation^a^	Matched controls^a^	*P_uncorr_* ^b^	*P_FDR_* ^c^	Cohen *d*
**Cognitive domains**					
Attention/executive dysfunction, z	−0.058 ± 0.851 (787)	0.039 ± 0.820 (1817)	0.006	**0.012** *	0.12
Information processing speed, z	−0.039 ± 0.720 (836)	0.031 ± 0.722 (1895)	0.051	0.067	0.1
Memory, z	−0.007 ± 0.570 (779)	0.012 ± 0.556 (1749)	0.515	0.515	0.03
Reasoning, z	−0.085 ± 0.837 (843)	0.049 ± 0.838 (1893)	<0.001	**<0.001** ***	0.16
**Cortical gray matter**					
Cortical thickness, mm	2.367 ± 0.087 (1293)	2.376 ± 0.088 (2594)	0.001	**0.004** **	0.10
Gyrification index	27.623 ± 0.501 (1293)	27.629 ± 0.517 (2594)	0.783	0.830	0.01
Sulcal depth, mm	9.134 ± 0.471 (1293)	9.135 ± 0.472 (2594)	0.860	0.860	0.00
Fractal dimension	2.613 ± 0.024 (1293)	2.614 ± 0.024 (2594)	0.183	0.274	0.04
Gray matter volume	0.458 ± 0.048 (1293)	0.461 ± 0.047 (2594)	0.014	**0.032** *	0.06
Fractional anisotropy	0.144 ± 0.093 (1335)	0.144 ± 0.089 (2683)	0.389	0.539	−0.01
Mean diffusivity, 10^‐3^ mm^2^/s	1.083 ± 0.080 (1335)	1.083 ± 0.076 (2683)	0.044	0.072	−0.01
Isotropic volume fraction	0.254 ± 0.041 (1335)	0.253 ± 0.039 (2683)	0.003	**0.008** **	−0.03
Neurite density index	0.343 ± 0.024 (1335)	0.342 ± 0.021 (2683)	0.779	0.829	−0.02
Orientation dispersion	0.455 ± 0.018 (1335)	0.455 ± 0.017 (2683)	0.496	0.595	−0.00
**White matter**					
Fractional anisotropy (FA)	0.408 ± 0.016 (1335)	0.409 ± 0.018 (2683)	0.007	**0.017** *	0.05
Mean diffusivity (MD), 10^‐3^ mm^2^/s	0.810 ± 0.033 (1335)	0.808 ± 0.33 (2683)	<0.001	**0.003** **	−0.06
Isotropic volume fraction	0.086 ± 0.014 (1335)	0.085 ± 0.014 (2683)	<0.001	**<0.001** ***	−0.08
Neurite density index	0.554 ± 0.028 (1335)	0.554 ± 0.027 (2683)	0.350	0.484	0.00
Orientation dispersion	0.256 ± 0.011 (1335)	0.255 ± 0.012 (2683)	0.045	0.074	−0.04
White matter hyperintensity load, %	0.005 ± 0.005 (1215)	0.004 ± 0.004 (2645)	<0.001	**<0.001** ***	−0.15
Peak width of skeletonized mean diffusivity, 10^‐3^ mm^2^/s	0.257 ± 0.052 (1335)	0.251 ± 0.06 (2683)	<0.001	**<0.001** ***	−0.10

Abbreviations: mm, millimeters; SD, standard deviation; z, *z* score.

Bold text indicates statistical significance.

^a^ Presented as mean ± SD (N).

^b^ Uncorrected *P* values of analyses of covariance, adjusted for age, sex, education, and cardiovascular risk factors.

^c^ False discovery rate‐corrected *P* values of analyses of covariance, adjusted for age, sex, education, and cardiovascular risk factors.

*P < 0.05, **P < 0.01, ***P < 0.001.

### 3.3 Global analysis of imaging markers

To test for group differences in imaging markers averaged across the entire cortical gray matter or white matter, we conducted ANCOVAs, adjusted for sex, age, education, and cardiovascular risk factors (see Table 2).

Regarding markers of cortical macrostructure, subjects with AF showed a lower cortical thickness and gray matter volume compared to the control group (mean ± SD, cortical thickness [mm]: 2.367 ± 0.087 vs. 2.376 ± 0.088, Cohen *d* = 0.10, *P_FDR _* = 0.004; gray matter volume: 0.458 ± 0.048 vs. 0.461 ± 0.047, Cohen *d* = 0.06, *P_FDR _* = 0.032). There were no significant differences between the groups concerning cortical folding geometry.

In our analysis of global cortical microstructure markers, the AF group showed a higher isotropic volume fraction (0.254 ± 0.041 vs. 0.253 ± 0.039, Cohen *d* = −0.03, *P_FDR _* = 0.008), suggesting a heightened presence of extracellular free water within the cortical tissue. Other markers including FA, MD, orientation dispersion, and neurite density in the gray matter were comparable between the groups.

Our investigation of mean skeletonized markers of white matter microstructure revealed both a lower global FA and a higher MD in the AF group compared to the control group (mean ± SD, FA: 0.408 ± 0.016 vs. 0.409 ± 0.018, Cohen *d* = 0.05, *P_FDR _* = 0.017; MD [10^−3^ mm^2^/s]: 0.810 ± 0.033 vs. 0.808 ± 0.33, Cohen *d* = −0.06, *P_FDR _* = 0.003). This reflects lower directionality of water diffusion with overall higher bulk diffusivity. Additionally, the AF group displayed a higher global isotropic volume fraction in the white matter (mean ± SD, 0.086 ± 0.014 vs. 0.085 ± 0.014, Cohen *d* = −0.08, *P_FDR _* = 0.008), indicating a higher proportion of extracellular free water in the white matter. Global orientation dispersion and neurite density showed no significant group differences.

Evaluating white matter markers related to small vessel pathology, in subjects with AF we observed a higher WMH load (mean ± SD [%], 0.005 ± 0.005 vs. 0.004 ± 0.004, Cohen *d* = −0.15, *P_FDR _* < 0.001) and a higher PSMD (mean ± SD [10^−3^ mm^2^/s], 0.257 ± 0.052 vs. 0.251 ± 0.06, Cohen *d* = −0.10, *P_FDR _* < 0.001). These results remained stable when additionally controlling for AF comorbidities (Table S3).

### 3.4 Mediation analysis

**FIGURE 2:**
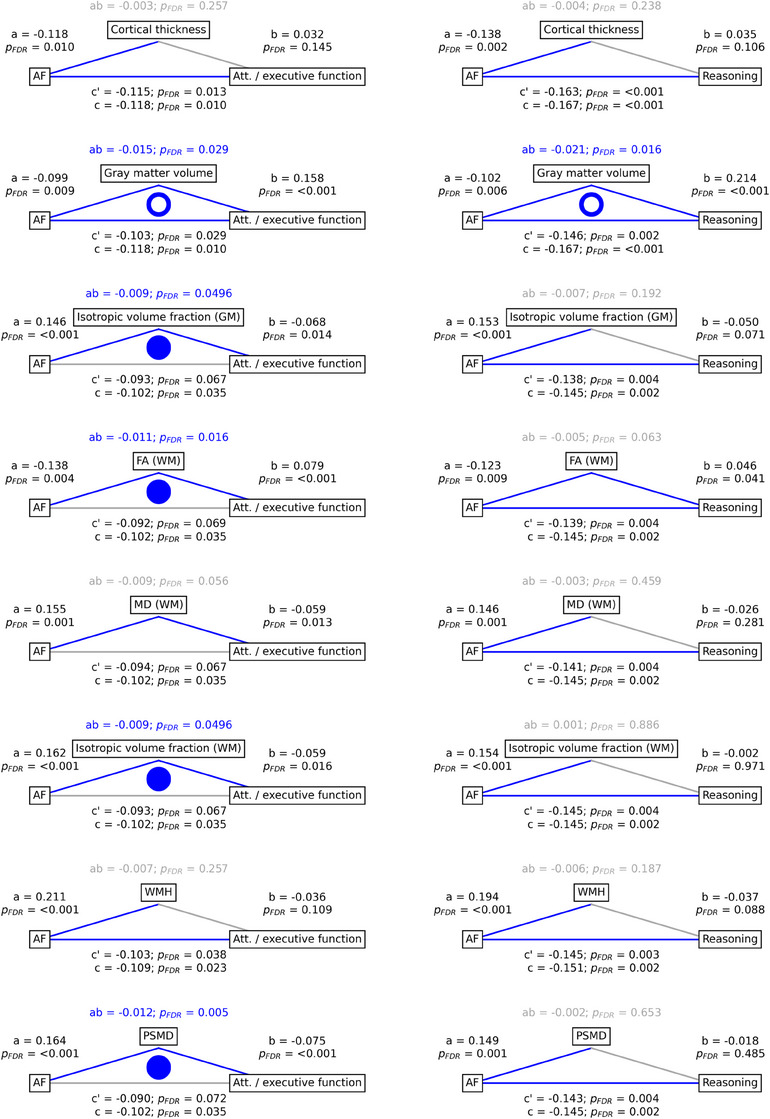
Mediation analysis. Mediation effects of global imaging markers on the relationship between AF and attention/executive function as well as reasoning. Path plots display standardized effects and *P* values: (a) AF to imaging marker, (b) imaging marker to cognitive score, (ab) indirect effect, (c') direct effect, and (c) total effect. Significant paths are highlighted in blue; non‐significant in light gray. If a relationship is significantly mediated, that is, the indirect effect ab was significant and the direct effect c' was reduced or non‐significant compared to the total effect c, the text for ab is highlighted in blue. Only imaging markers with significant differences between AF and control groups are shown. The left path plots show results regarding attention/executive function, while the right plots depict those of reasoning. For path plots on all global imaging markers refer to supplementary Figure S3 in supporting information. Dots in the center of the path plots represent significant mediation effects. An empty dot indicates a partial mediation, a full dot indicates full mediation. Abbreviations: AF, atrial fibrillation; FA, fractional anisotropy; GM, gray matter; MD, mean diffusivity; PSMD, peak width of skeletonized mean diffusivity; WM, white matter; WMH, white matter hyperintensity.

To investigate whether global imaging markers mediate the relationship between AF and cognitive domain performance, we performed a mediation analysis (Figure 2).

The relationship between AF and attention/executive function was found to be partially mediated by gray matter volume (ab = −0.015, *P_FDR _* = 0.029; c’ = −0.103, *P_FDR_* 0.029; c = −0.118, *P_FDR_* = 0.010) and fully mediated by gray matter isotropic volume fraction (ab = −0.009, *P_FDR _* = 0.00496; c’ = −0.093, *P_FDR_* 0.067; c = −0.102, *P_FDR_* = 0.035), white matter FA (ab = −0.011, *P_FDR _* = 0.016; c’ = −0.092, *P_FDR_* 0.069; c = −0.102, *P_FDR_* = 0.035), white matter isotropic volume fraction (ab = −0.009, *P_FDR _* = 0.0496; c’ = −0.093, *P_FDR_* 0.067; c = −0.102, *P_FDR_* = 0.035), and PSMD (ab = −0.012, *P_FDR _* = 0.005; c’ = −0.090, *P_FDR_* 0.072; c = −0.102, *P_FDR_* = 0.035).

The relationship between AF and reasoning was partially mediated by gray matter volume (ab = −0.021, *P_FDR _* = 0.016; c’ = −0.146, *P_FDR_* 0.002; c = −0.167, *P_FDR_* < 0.001). For path plots showing mediation analysis results of all global imaging markers refer to Figure S3 in supporting information. Scatter plots of the relationship between imaging markers and cognitive measures are shown in Figures S4–S6 in supporting information.

### 3.5 Regional distribution of cortical differences in macro‐ and microstructure

To detect spatial patterns of brain structural alterations, we performed ROI‐wise analyses of gray and white matter imaging markers and TBSS.

Comparisons of Schaefer400‐parcellated cortical thickness and gray matter volume revealed significant differences between the AF group and matched controls (Figure 2). Individuals with AF exhibited lower cortical thickness in the primary sensorimotor and visual cortices, that is, M1, S1, V1, as well as orbitofrontal and lateral prefrontal areas, the posterior insula, the superior temporal gyrus, bilaterally. Furthermore, individuals with AF showed lower bilateral gray matter volume in M1, S1, V1, as well as the middle and inferior temporal gyrus. Variations in cortical folding metrics were either restricted to specific parcels or were non‐significant.

**FIGURE 3:**
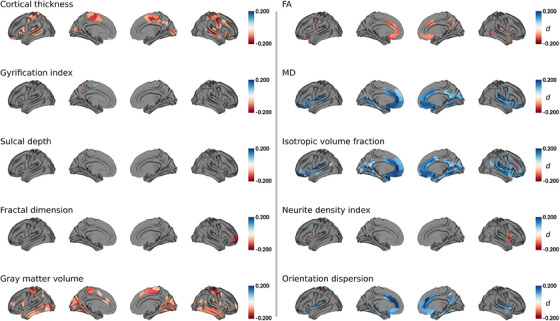
Group comparison of cortical macro‐ and microstructural indices. Regions of interest that significantly differed between groups are highlighted by colors encoding Cohen *d*: AF individuals < matched controls, red; AF individuals > matched controls, blue. Abbreviations: *d* , Cohen *d*; FA, fractional anisotropy; MD, means diffusivity.

Comparisons of cortical microstructural measures revealed significant differences between the AF group and matched controls in the bilateral anterior cingulate, insula, medial prefrontal cortex, medial temporal lobe, and precuneus (Figure 3). These regions exhibited higher isotropic volume fraction and to varying degrees a lower FA and higher MD and orientation dispersion. Localized lower neurite density was found in in the anterior insular and adjacent zones. Cumulatively, the results identify microstructural irregularities in the affected areas, with alterations in the extracellular compartment. For statistical comparisons of all cortical effect maps please refer to Figure S7 in supporting information.

### 3.6 Regional distribution of structural differences in the white matter

**FIGURE 4:**
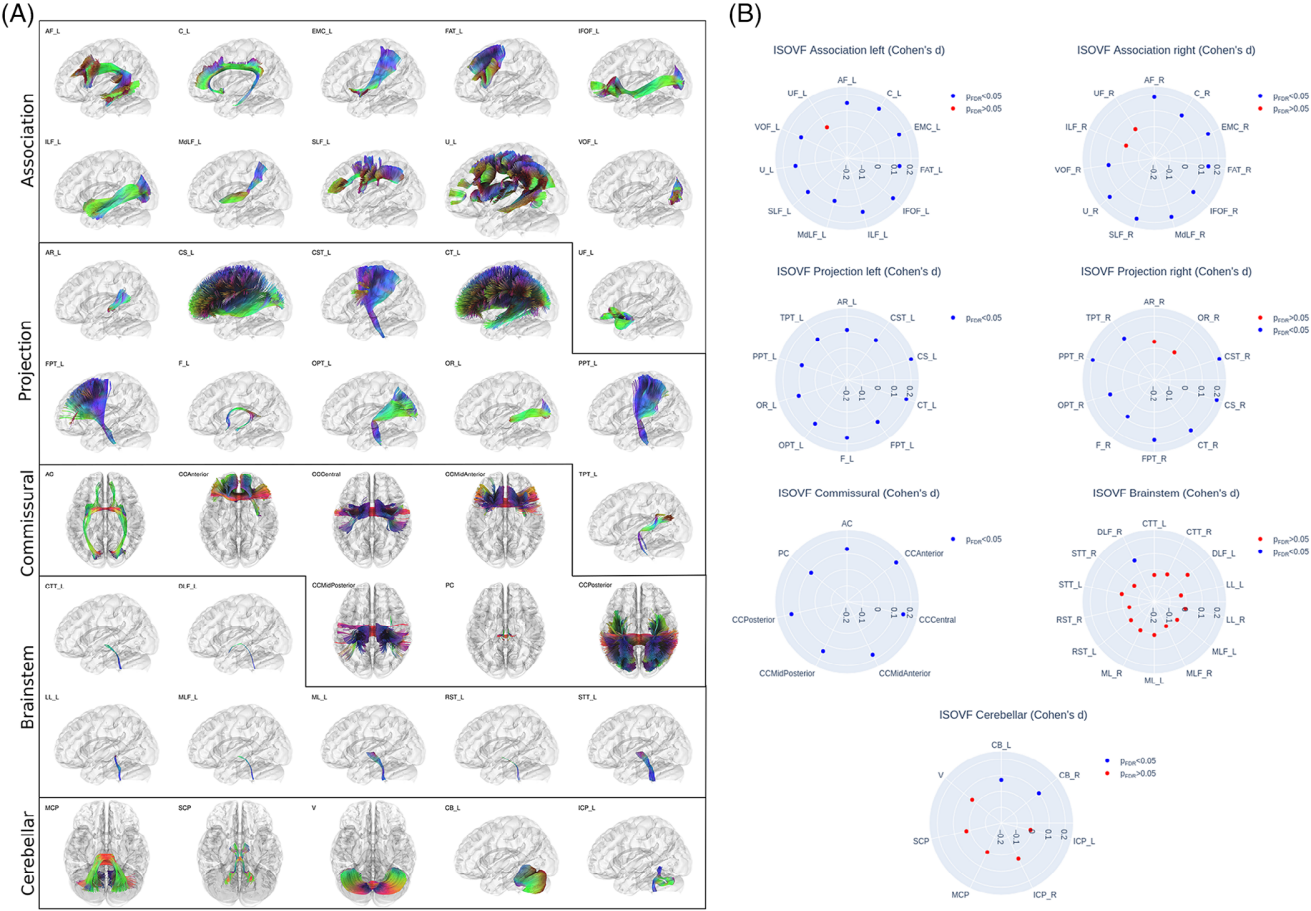
Group comparison of tract‐level isotropic volume fraction. Left panel: anatomical depiction of the white matter tracts investigated, categorized into association, projection, commissural, brainstem, and cerebellar tracts. For paired tracts only left side examples are visualized. Right panel: radar plots represent the group differences (Cohen *d*) for isotropic volume fraction in each tract, with blue dots indicating significant differences and red dots marking non‐significant differences. Each radar plot corresponds to one of the tract groups highlighted in the left panel. Tract abbreviations: Association tracts—AF, arcuate fascicle; C, cingulate; EMC, extreme capsule; FAT, frontal aslant tract; IFOF, inferior fronto‐occipital fasciculus; ILF, inferior longitudinal fasciculus; MdLF, middle longitudinal fasciculus; SLF, superior longitudinal fasciculus; U, U‐fibers; UF, uncinate fasciculus; VOF, vertical occipital fasciculus. Projection tracts – AR, acoustic radiation; CS, corticostriatal pathway; CST, corticospinal tract; CT, corticothalamic pathway; F, fornix; FPT, frontopontine tract; OPT, occipitopontine tract; OR, optic radiation; PPT, parietopontine tract; TPT, temporopontine tract. Commisural tracts – AC, anterior commissure; CC, corpus callosum; PC, posterior commissure. Brainstem tracts – CTT, central tegmental tract; DLF, dorsal longitudinal fasciculus; LL, lateral lemniscus; ML, medial lemniscus; MLF, medial longitudinal fasciculus; RST, rubrospinal tract; STT, spinothalamic tract. Cerebellar tracts – CB, cerebellum; ICP, inferior cerebellar peduncle; MCP, middle cerebellar peduncle; SCP, superior cerebellar peduncle; V, vermis. ISOVF, isotropic volume fraction.

Analysis of microstructural measures in predefined anatomical white matter tracts disclosed widespread differences for FA, MD, isotropic volume fraction, and orientation dispersion between the AF group and matched controls. Differences were found in multiple association, commissural, and projection tracts, but only few brainstem and cerebellar tracts (corresponding details on all tracts investigated are illustrated in Figure 4 and Figures S8–S11 in supporting information): higher isotropic volume fraction in 47 (67.1%) tracts, lower FA was found in 43 (61.4%) tracts, higher MD in 47 (67.1%) tracts, and higher orientation dispersion in 25 (35.7%) tracts. There were no significant differences for tract‐level neurite density. Taken together, this imaging profile indicates extensive microstructural abnormalities throughout the white matter, with an emphasis on changes in the extracellular compartment.

**FIGURE 5:**
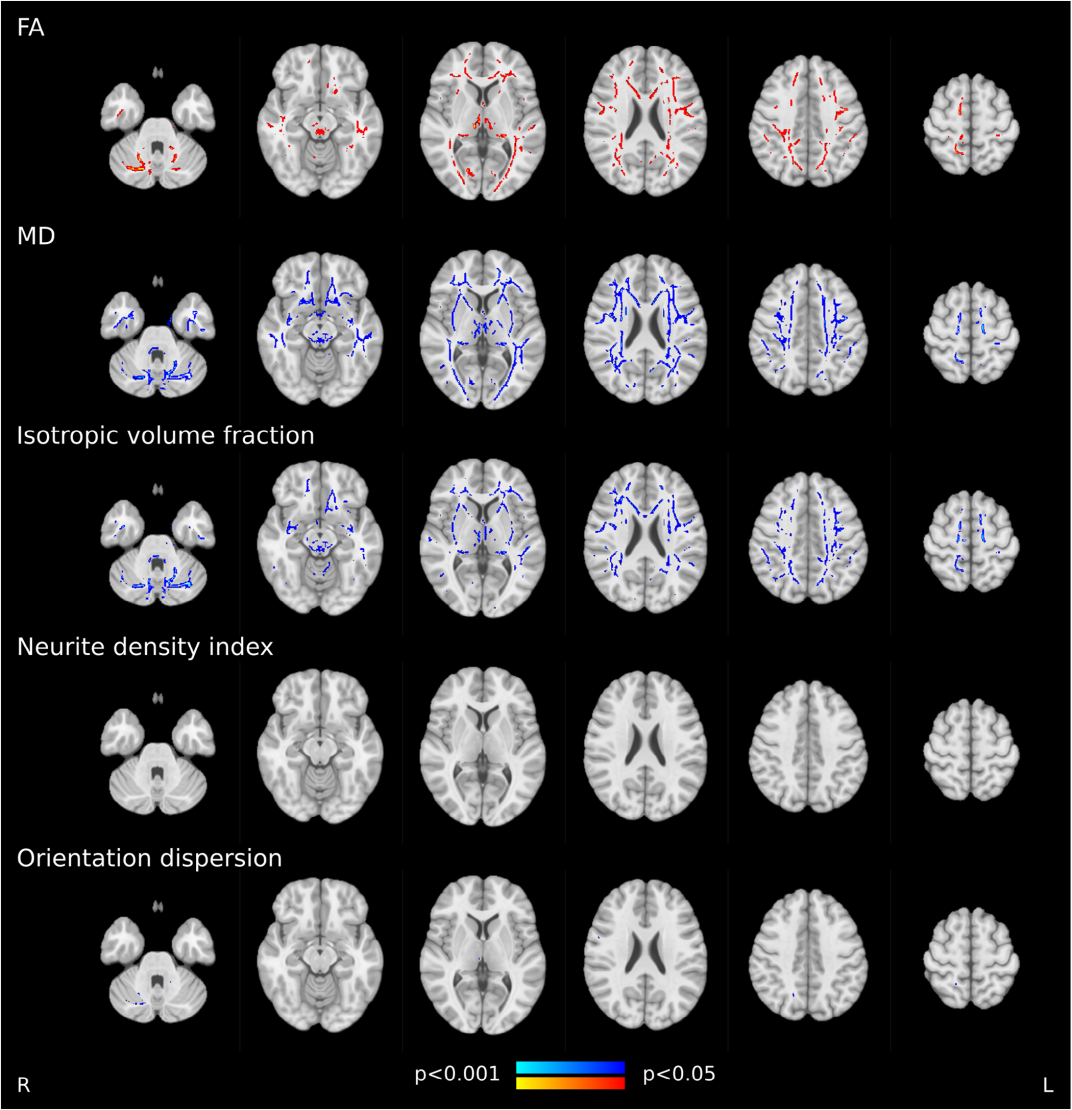
Tract‐based spatial statistics of white matter imaging markers. Skeleton voxels that significantly differed between groups are highlighted by colors: AF individuals < matched controls, red; AF individuals > matched controls, blue. Abbreviations: FA, fractional anisotropy; MD, mean diffusivity.

We complemented the tract‐level approach by TBSS, that is, voxel‐wise statistics on the entire white matter skeleton. Corresponding results echoed tract‐level findings by demonstrating widespread lower FA as well as higher MD and isotropic volume fraction in the white matter skeleton of AF individuals encompassing all brain lobes (Figure 5). Group differences of neurite density and orientation dispersion were non‐significant or spatially limited to few isolated voxel groups, respectively.

## 4 DISCUSSION

**FIGURE 6:**
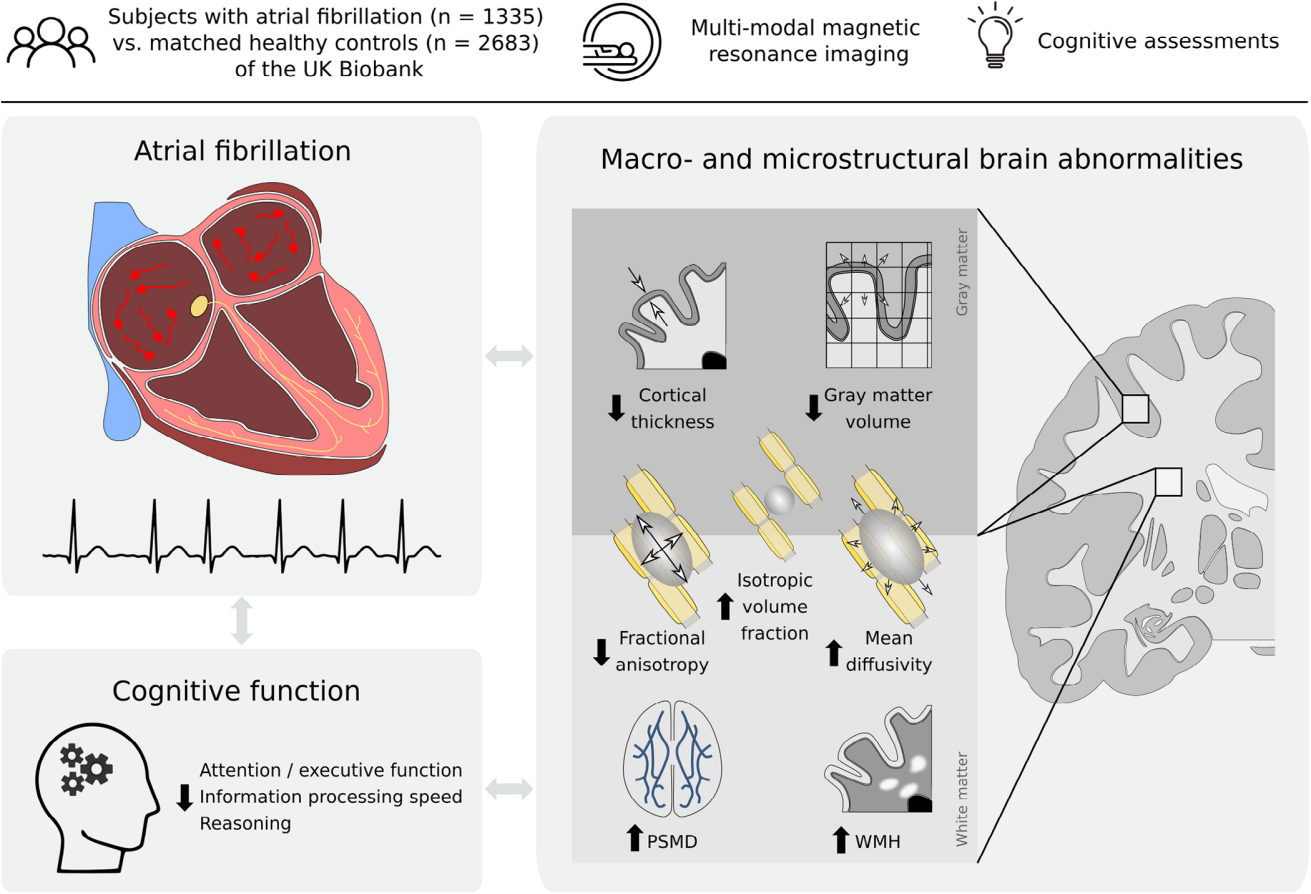
Graphical abstract. Abbreviations: PSMD, peak width of skeletonized mean diffusivity; WMH, white matter hyperintensity.

In a cohort of AF individuals without prior stroke or dementia, we examined cross‐domain cognitive performance and multimodal neuroimaging of brain macro‐ and microstructure. Our analysis revealed lower performance in the cognitive domains of attention/executive function and reasoning in individuals with AF. These cognitive deficits were accompanied by three main imaging findings: AF was associated with (1) macrostructural abnormalities of the cerebral cortex, including lower cortical thickness and gray matter volume predominantly in M1, S1, V1, as well as orbitofrontal, lateral prefrontal, posterior insular, and temporal cortices; (2) microstructural cortical abnormalities of higher isotropic volume fraction in the anterior cingulate, insula, medial prefrontal cortex, medial temporal lobe, and precuneus; and (3) widespread microstructural anomalies in the cerebral white matter, marked by lower FA, higher MD, and isotropic volume fraction and higher burden of the small vessel disease markers WMH load and PSMD. Crucially, imaging markers statistically mediated the relationship of AF and cognitive domain performance. A graphical abstract summarizing the results is included as Figure 6. Overall, our findings identify a complex profile of structural brain changes that may provide novel links between AF and cognitive performance.

### 4.1 AF is independently linked to cognitive deficits

Numerous prospective studies have observed an association between AF and cognitive disorders, spanning mild cognitive impairment to Alzheimer's disease and vascular dementia.^40^, ^41^, ^42^ We examined the cognitive performance differences linked to AF in a population‐based sample of stroke‐ and dementia‐free individuals. To isolate the cognitive effects attributable to AF, we matched relevant confounders and statistically adjusted for them as well as excluded subjects with prevalent neurological conditions. We observed a lower performance in cognitive domain scores of attention/executive function and reasoning implying a significant association of AF and cognitive impairment independent of confounders. These results remained robust when additionally controlling for the prevalence of common AF comorbidities including atherosclerotic heart disease and congestive heart failure. There were no differences in domain scores of information processing speed and memory performance; however, on the individual cognitive test level, the lower performance on the Digit Symbol Substitution Test indicates reduced speed of information processing. Our observations highlight deficits particularly in executive function while memory was not affected, echoing findings from previous population‐based studies.^43^, ^44^, ^45^ Other studies reported associations across different cognitive domains, which led to the notion that AF impairs all major cognitive domains.^6^ We speculate that the differential associations of AF and cognition depend on stage‐specific brain structural changes. Specifically, initial localized variations in tissue macro‐ and microstructure may be linked to focal cognitive deficits. As the disease advances, this pathology might spread to other regions, leading to widespread cognitive decline across multiple domains. Taken together, our results corroborate previous analyses reporting cognitive deficits in AF.

### 4.2 AF is associated with altered cortical morphology

AF is considered to compromise brain health via complex, interacting effects on cerebral vasculature and parenchyma.^7^ To delineate these impacts, we investigated advanced neuroimaging markers of both gray and white matter integrity. Our results revealed abnormalities in both macro‐ and microstructural markers within the AF cohort.

We found altered cortical thickness and gray matter volume in AF individuals indicating neurodegenerative effects. AF might cause a decline in cortical thickness and gray matter volume by driving multiple vascular and inflammatory mechanisms, which impede proper blood flow and oxygenation, thus accelerating neuronal tissue loss. Although our study is the first to investigate markers of cortical macrostructure in AF, a negative correlation between AF and overall brain volume has been highlighted in previous reports, reinforcing our observations.^6^, ^46^, ^47^, ^48^, ^49^ On a regional scale, we found gray matter macrostructural differences to be localized bilaterally to primary sensorimotor and visual areas, orbitofrontal and lateral prefrontal cortices, the posterior insula, and the temporal lobe. We interpret the relative symmetry of effects as indicative of vascular contributions, given the topological symmetry of vascular supply. Notably, the observed regions coincide with effect maps of prior studies on cortical macrostructural effects of small vessel pathology in mild vascular cognitive impairment as well as in stroke.^50^, ^51^, ^52^ Thus, we hypothesize that the AF‐induced injury of the cerebral cortex is relevantly influenced by small vessel disease, which in turn can lead to vascular cognitive deficits, offering a plausible connection between AF and cognitive decline, as also discussed for other cardiac diseases.^53^

### 4.3 AF relates to extracellular free water increases in gray and white matter

Turning to MRI markers of microstructural integrity, the AF group prominently displayed elevated isotropic volume fraction in the gray and white matter, indicative of an increase in extracellular free water. Regional alterations in isotropic volume fraction were accompanied by changes in FA, denoting decreased water diffusion directionality; MD, indicating heightened overall water diffusivity; and orientation dispersion; implying fiber configuration shifts. Importantly, the neurite density index, a marker of cellular tissue, remained largely unchanged, suggesting preserved cellular and neurite integrity. These findings expand on a previous analysis showing globally reduced directionality of water diffusion in the white matter of individuals with AF.^49^ Taken together, the observed alterations in the different parameters derived from DWI likely reflect increased amounts of extracellular free water alongside subtle cellular abnormalities like demyelination and dispersion of neurite orientations. We hypothesize that higher extracellular free water and neurite dispersion in AF could stem from blood–brain barrier leakage and inflammation‐driven osmotic shifts of water from blood to the extracellular space. A detailed discussion of results regarding regional differences of cortical microstructure is presented in Text S2 in supporting information.

Our findings further highlight elevated global markers of small vessel pathology, specifically WMH load and PSMD, in subjects with AF. Hence, we argue that the microstructural effects in gray and white matter are relevantly attributable to AF‐related small vessel pathology. This hypothesis aligns with previous reports indicating that extracellular free water, as we observed in AF, is the primary contributor to tissue diffusion changes in small vessel pathology.^54^, ^55^ Another study in a memory clinic cohort found that tissue free water fully mediated the relationship between cardiac biomarkers and a 5‐year longitudinal cognitive decline, positioning the biomarker as a centerpiece for characterizing heart–brain interactions.^56^

### 4.4 Brain structural differences mediate the link of AF and cognition

Integrating the observed group differences in cognitive and imaging markers, we conducted a mediation analysis to statistically assess brain structure as a potential pathomechanistic link between AF and cognitive function. We found that the association between AF and cognitive function was significantly mediated by structural imaging markers. Specifically, the relationship between AF and attention/executive function was mediated by gray matter volume, gray matter isotropic volume fraction, white matter FA, white matter isotropic volume fraction, and PSMD and between AF and reasoning by gray matter volume. These results suggest that AF's link to cognitive function depends to a relevant extent on differences in these imaging markers, underscoring the significance of macrostructural and microstructural brain changes in AF‐related cognitive sequelae.

### 4.5 A unifying hypothesis to explain brain imaging correlates of AF in the normal population

Drawing upon a comprehensive analysis of brain imaging correlates associated with AF, our research converges on a unifying hypothesis: small vessel pathology stands out as a key mechanism connecting AF to cognitive decline in individuals unaffected by stroke or dementia. This assertion is supported by several key observations. First, our results underscore the involvement of executive dysfunction and information processing speed both being key cognitive correlates of small vessel disease, while memory was not affected in our cohort of AF subjects.^57^ Second, the observed macro‐ and microstructural differences in the cortex echo patterns reflecting various progression stages of vascular cognitive impairment. Last, the widespread alterations in the white matter mirror a neuroimaging profile consistent with small vessel disease, accompanied by heightened WMH load and PSMD.^58^

Our findings not only shed light on the intricate relationships among AF, cognitive decline, and neuroanatomical changes, but they also hint at potential avenues of clinical use. The definitive role of AF treatments, particularly in precluding cognitive comorbidities, is yet to be firmly established. However, there is evidence suggesting that interventions targeting AF might reverse prothrombogenic and proinflammatory cascades, as well as recovering cerebral perfusion.^59^ Notably, there have been promising outcomes related to oral anticoagulation, pointing toward enhanced cognitive trajectories.^60^, ^61^ Preliminary observational datasets also hint at the potential cognitive benefits associated with rhythm control strategies, either pharmacological or non‐pharmacological.^62^ Moving forward, leveraging neuroimaging could enable patient‐tailored therapeutic interventions, facilitating the identification of subgroups at risk of cognitive disorders likely to reap the most substantial benefits.

### 4.6 Strengths and limitations

A strength of our study is the use of large‐scale clinical and multimodal MRI data, offering a detailed view of the link between AF and brain health. Nonetheless, we acknowledge some limitations of our work. First, due to the indirect nature of the performed imaging–phenotype association analyses, our findings cannot firmly establish causal links and should be regarded as hypothesis generating. Second, despite controlling for cardiovascular risk factors through matching and assessing the effects of comorbidities in our statistical models, we cannot entirely rule out the influence of AF comorbidities on the relationships among AF, brain structure, and cognition. Furthermore, other variables potentially contributing to cognitive variance were not accounted for including AF subtypes, depression status, obesity, and medication. Taken together, longitudinal and experimental studies are needed that expand on our findings to further discern AF effects on brain health. Future studies including measures to further characterize AF by comprehensive analysis of cardiac MRI and echocardiography might provide additional clues on heart–brain interactions.

### 4.7 Conclusion

Our comprehensive exploration of a large cohort of individuals with AF unveiled associations among AF, structural brain changes, and cognitive decline, notably in individuals without a history of stroke or dementia. Our research emphasizes small vessel pathology as a potential mechanism associating AF with cognitive deficits. We observed cognitive deficits and macro‐ and microstructural brain alterations mirroring those observed in vascular cognitive impairment. As this research field progresses, harnessing neuroimaging could pave the way for individualized therapeutic strategies.

## CONFLICT OF INTEREST STATEMENT

G.T. has received fees as consultant or lecturer from Acandis, Alexion, Amarin, Bayer, Boehringer Ingelheim, BristolMyersSquibb/Pfizer, Daichi Sankyo, Portola, and Stryker outside the submitted work. P.K. reports holding patent WO2015140571 on atrial fibrillation therapy, licensed to the University of Birmingham, and patent WO2016012783 on markers for atrial fibrillation, licensed to the University of Birmingham. The remaining authors declare no conflicts of interest. Author disclosures are available in the supporting information.

## CONSENT STATEMENT

Written informed consent was obtained from all participants investigated in this work.

## Supporting information

Supporting Information

Supporting Information
