# Supplementary material for: Mapping the interplay of atrial fibrillation, brain structure, and cognitive dysfunction
Source: Alzheimers Dement. 2024 Jun 5;20(7):4512–26. doi: 10.1002/alz.13870 (PMC11247702; doi:10.1002/alz.13870)
Supplement: Supplementary file 1 — Supporting Information [file ALZ-20-4512-s002.pdf]

Mapping the Interplay of Atrial Fibrillation, Brain Structure and Cognitive Dysfunction

–

Supporting Information

Marvin Petersen, MD[a]; Céleste Chevalier, MD[b,c]; Felix L. Naegele, MD[a]; Thies Ingwersen, MD[a]; Amir Omidvarnia[d,e], PhD; Felix Hoffstaedter[d,e], PhD; Kaustubh Patil[d,e], PhD; Simon B. Eickhoff[d,e], MD; Renate B. Schnabel, MD[b,c]; Paulus Kirchhof[b,c], M.D; Eckhard Schlemm, MBBS, PhD[a]; Bastian Cheng, MD[a]; Götz Thomalla, MD[a]; Märit Jensen, MD[a]

[a]Department of Neurology, University Medical Center Hamburg-Eppendorf, Hamburg, Germany

[b]Department of Cardiology, University Heart and Vascular Center, Hamburg, Germany

[c]DZHK (German Center for Cardiovascular Research), partner site Hamburg/Kiel/Luebeck, Germany

[d]Institute for Systems Neuroscience, Medical Faculty, Heinrich-Heine University Düsseldorf, Düsseldorf, Germany

[e]Institute of Neuroscience and Medicine, Brain and Behaviour (INM-7), Research Center Jülich, Jülich, Germany

Content

Methods ... 3

Table S1 – Excluded diagnoses based on non-cancer illness information ... 3

Text S1 - Image processing ... 4

T1-weighted MRI ... 4

Diffusion-weighted MRI ... 5

Fluid-attenuated inversion recovery MRI ... 7

Quality assurance ... 7

Results ... 8

Figure S1 - Sample selection flowchart ... 8

Figure S2 – Matching results visualized as balance plot ... 9

Table S2 – AF comorbidities ... 10

Table S3 – Group comparisons controlling for AF comorbidities ... 11

Table S4 – Group comparisons of individual cognitive tests ... 13

Figure S3 – Mediation analysis results ... 14

Figure S4 – Regression analysis: cognition ~ cortical macrostructural imaging markers ... 16

Figure S5 – Regression analysis: cognition ~ cortical microstructural imaging markers ... 17

Figure S6 – Regression analysis: cognition ~ white matter imaging markers ... 18

Figure S7 – Cortical effect maps comparison ... 19

Figure S8 - Group comparison of tract-level fractional anisotropy (FA) ... 20

Figure S9 - Group comparison of tract-level mean diffusivity (MD) ... 21

Figure S10 - Group comparison of tract-level neurite density index ... 22

Figure S11 - Group comparison of tract-level orientation dispersion ... 23

Discussion ... 24

Text S2 – Microstructural abnormalities map to specific cortical networks ... 24

References ... 26

Methods

Table S1 – Excluded diagnoses based on non-cancer illness information

ID	Diagnosis
1081	stroke
1082	transient ischaemic attack (tia)
1083	subdural haemorrhage/haematoma
1086	subarachnoid haemorrhage
1240	neurological injury/trauma
1244	infection of nervous system
1245	brain abscess/intracranial abscess
1246	encephalitis
1247	meningitis
1258	chronic/degenerative neurological problem
1261	multiple sclerosis
1262	parkinsons disease
1263	dementia/alzheimers/cognitive impairment
1264	epilepsy
1266	head injury
1408	alcohol dependency
1409	opioid dependency
1410	other substance abuse/dependency
1434	other neurological problem
1491	brain haemorrhage
1583	ischaemic stroke
1626	fracture skull / head

Text S1 - Image processing

T1-weighted MRI

T1w image processing was performed in CAT12.[1] CAT12 processing can be divided into 2 streams: (1) surface-based morphometry (SBM), and (2) voxel-based morphometry (VBM). Together, SBM and VBM are imaging methods that enable the identification and characterization of macrostructural brain abnormalities.

Surface-based morphometry

SBM results in vertex-level measures of cortical thickness and cortical folding, i.e., point-wise estimates of cortical geometry. Surface-based morphometry was performed in two stages: (1) surface creation and registration and (2) computation of morphometric estimates. In the surface creation phase, CAT12 utilized a projection-based thickness method to derive the initial cortical thickness and central surface. Subsequently, the central surface underwent refinement and topological defect correction, yielding final central, pial, and white matter surface meshes. These refined surfaces were then employed to re-estimate cortical thickness using the FreeSurfer thickness method, which assesses the width of the gray matter ribbon by measuring the distance between its inner and outer boundaries.[2] Furthermore, the final central surface served for the computation of three cortical folding metrics. The gyrification index was computed as the "smoothed absolute mean curvature", which involved averaging curvature values from each vertex of a spherical surface mesh within a 3 mm radius.[3] Larger negative gyrification index values signify sulci, while positive values represent gyri. Sulcal depth was computed as the distance between a point on the sulcal surface and the nearest point on the brain's convex hull, which envelopes the cortex's outermost (pial) surface.[4] Deeper sulcal depths indicate more pronounced sulci, while shallower depths suggest less pronounced ones. Fractal dimension was calculated as the slope of a logarithmic plot comparing surface area to the maximum l-value of the surface reconstruction (a measure of the bandwidth used to reconstruct the surface shape), with a higher FD indicating greater complexity and irregularity of cortical folding, and a lower FD denoting a smoother surface.[5] Cortical thickness was

smoothed using a 12mm kernel, while the cortical folding metrics were smoothed with a 20mm kernel.

Voxel-based morphometry

Voxel-based morphometry (VBM) results in voxel-wise estimates of gray matter volume. In CAT12, the VBM protocol include a tissue segmentation and spatial registration procedure. First, the T1w was denoised by applying a spatially adaptive non-local means (SANLM) filter. After the denoising, SPM's unified segmentation was performed, producing segmentation maps of multiple brain tissues, including a gray matter map. These gray matter segmentations capture the voxel-wise gray matter content. The tissue segmentations were registered to standardized MNI space templates using Geodesic Shooting.[6] The registration information was then incorporated into the gray matter segmentation by multiplying the deformation maps with the gray matter segmentation maps in a "modulation step." This adjustment accounts for volume alterations during spatial normalization, thereby estimating local gray matter volume in native space.[7] Finally, modulated gray matter volume maps were smoothed using a 6mm Gaussian kernel.

Diffusion-weighted MRI

Preprocessing

Analysis of diffusion-weighted MR images (DWI) was based on data already preprocessed by the UK Biobank. Details on UK Biobank preprocessing procedures can be found online (https://biobank.ctsu.ox.ac.uk/crystal/crystal/docs/brain_mri.pdf). In brief, DWI preprocessing included correction of eddy currents, head motion and outlier-slice removal using FSL's eddy as well as gradient distortion correction.[8]

Diffusion tensor imaging and Neurite Orientation Dispersion and Density Imaging

Fractional anisotropy (FA) and mean diffusivity (MD) were derived from diffusion tensors which were modelled based on preprocessed DWI using a least-squares fit.[9,10] FA and MD provide insights into the directionality and magnitude of water diffusion within tissues, respectively. In addition, Neurite Orientation Dispersion and Density Imaging (NODDI) was performed using

Accelerated Microstructure Imaging via Convex Optimization (AMICO, https://github.com/daducci/AMICO).[11,12] NODDI offers an assessment of the brain's microstructure beyond conventional DTI markers by capitalizing on a multi-compartment model. This model dissects the intricate features of neural tissue into distinct compartments. (1) The intracellular compartment, which showcases hindered or restricted diffusion, is quantified using the neurite density index (also intracellular volume fraction, ICVF). This measure essentially captures the proportion of space occupied by neuronal and glial cell bodies and their processes within a given voxel. (2) The extracellular compartment is characterized by isotropic diffusion, indicating equal diffusion in all directions. This is represented by measurement of isotropic volume fraction (ISOVF), which reflects the volume occupied by the space outside the cellular structures. Neurite orientations, a key aspect of neural tissue microstructure and organization, are described by orientation dispersion. This metric provides insights into the variability in the alignment of axons and dendrites within a voxel. Together, DTI and NODDI measures provide a detailed understanding of the underlying tissue microarchitecture, aiding in deciphering the intricate complexities of neural structures.

Tract-based spatial statistics

In order to derive skeletonized maps of each of the estimated diffusion parameters, we conducted tract-based spatial statistics (*TBSS*) utilizing the standardized FA template from FSL as the registration target.[13,14] Put briefly, individual FA images in template space got eroded to exclude non-brain voxels on the outer edge of the image. Next, a valid mask containing only the intersection of all subjects' brains was derived and used to mask the average of all previously eroded FA images. This mean FA image was subsequently used to derive a white matter skeleton. Next, all individual FA images were projected onto the mean FA skeleton. The resultant projection vectors were used to skeletonize all of the remaining diffusion metrics, i.e. MD and NODDI markers.

Peak width of skeletonized mean diffusivity

Peak width of skeletonized mean diffusivity (PSMD) was calculated based on standard procedures.[15] PSMD was calculated as the difference between the 95th and 5th percentile of MD values on the white matter skeleton in standard (MNI) space. A mask supplied by the developers was used to exclude white matter areas susceptible to partial volume effects of cerebrospinal fluid (https://github.com/miac-research/psmd/blob/main/skeleton_mask_2019.nii.gz).

Fluid-attenuated inversion recovery MRI

White matter hyperintensity segmentation

Precomputed WMH segmentation masks of the UK Biobank were used. To obtain them, FSL's *Brain Intensity AbNormality Classification Algorithm* (*BIANCA*)[16] was applied on FLAIR images and T1w images for white matter hyperintensity (WMH) segmentation. The WMH load was calculated as the WMH volume divided by the total intracranial volume calculated by CAT12.

Quality assurance

In order to minimize the influence of noise and artifacts, quality assurance (QA) of MRI data was conducted both quantitatively and qualitatively. For raw and processed T1w data, an image quality rating and sample homogeneity score were derived with CAT12. Visual QA of T1w imaging data was subsequently performed for individuals with an IQR and sample homogeneity score <0.8. For DWI, visual QA was performed on raw DWI and skeletonized diffusion indices in individuals with mean skeletonized diffusion metrics ± 2.5 standard deviations from the mean.

Results

Figure S1 - Sample selection flowchart

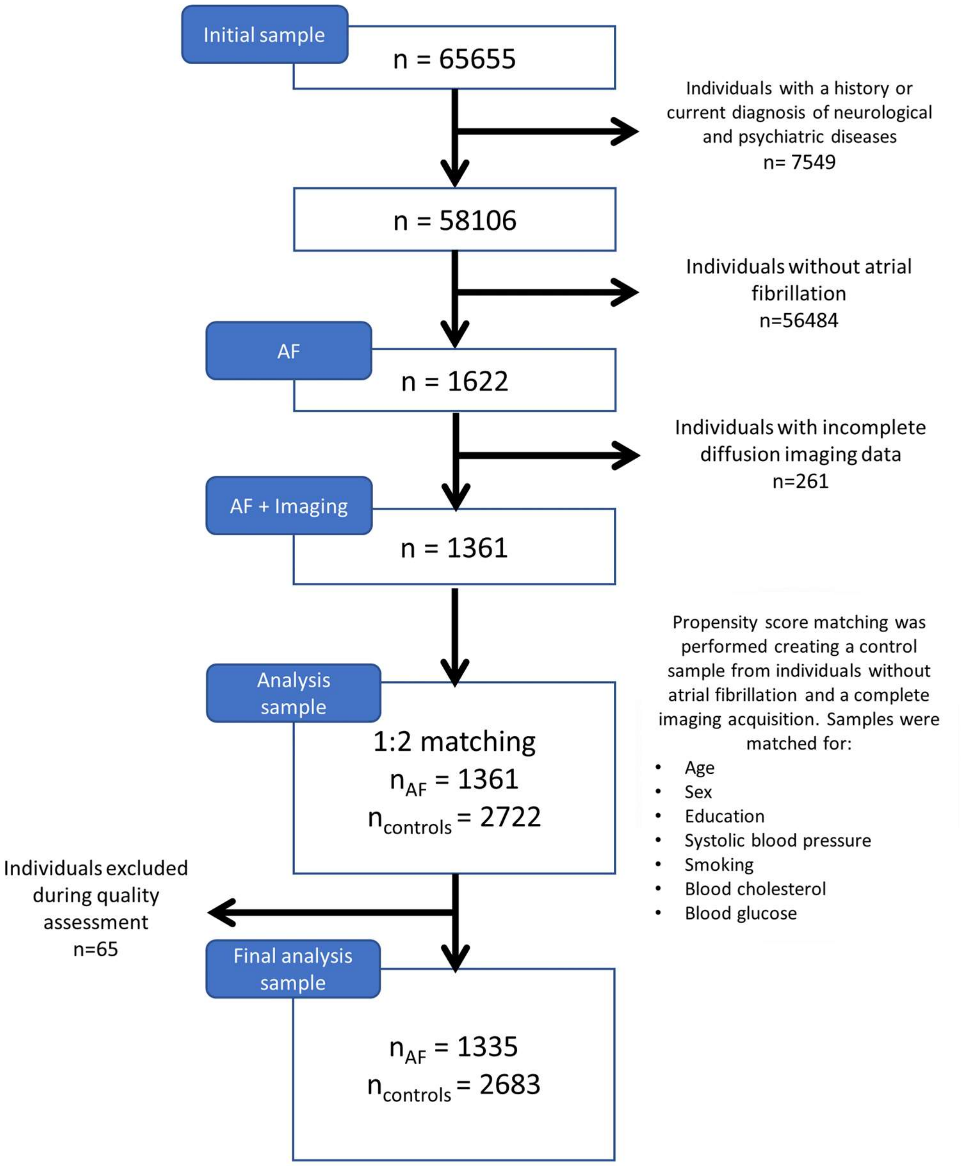

Figure S2 – Matching results visualized as balance plot

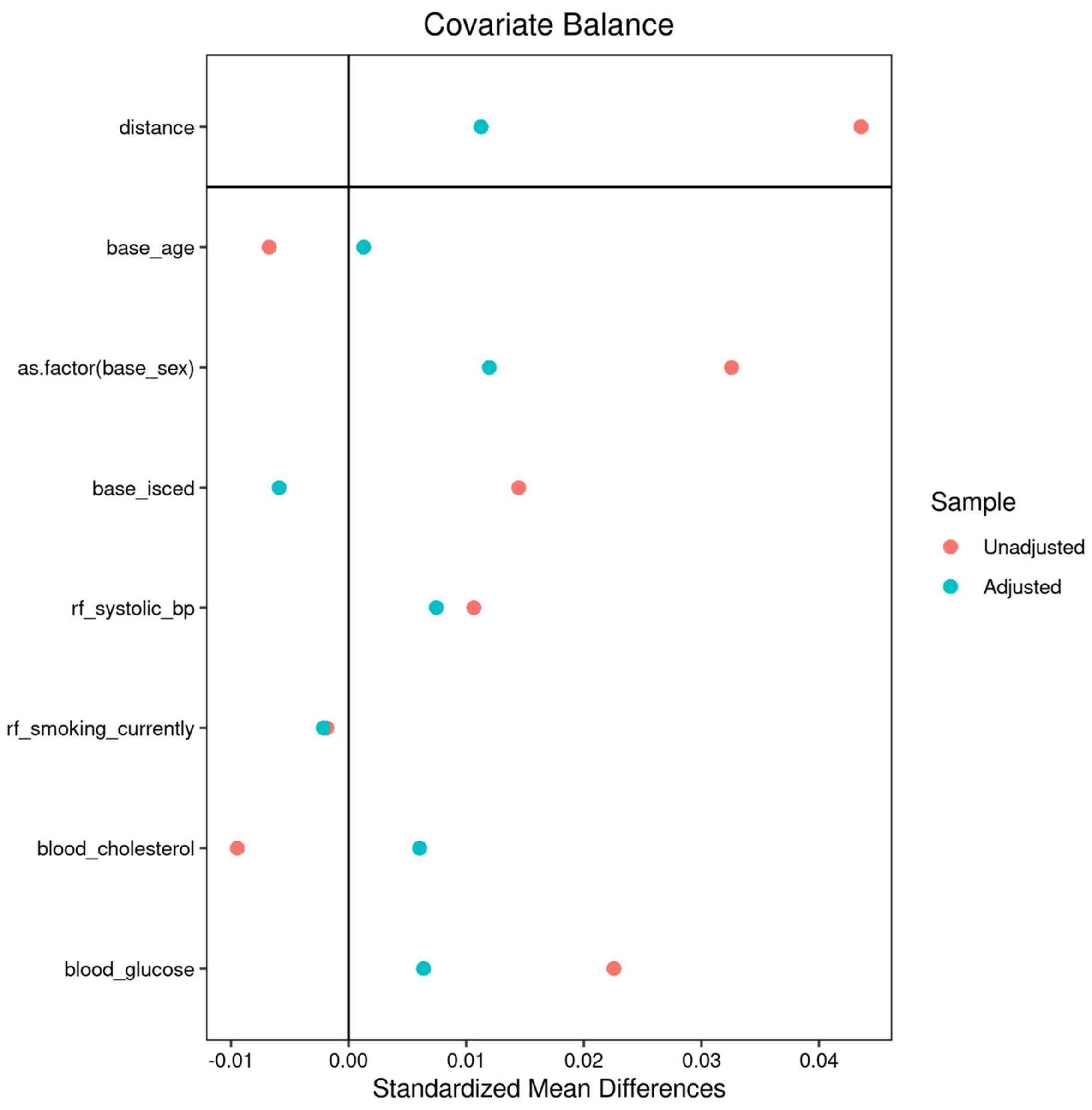

Standardized mean differences between the atrial fibrillation and healthy control group for each matching variable before (unadjusted, red) and after matching (adjusted, turquoise) are displayed. The closer the standardized mean difference is to zero, the more similar the groups are. Each matching variable is depicted separately, i.e., from top to bottom, age, sex, education, systolic blood pressure, smoking, blood cholesterol and blood glucose.

Table S2 – AF comorbidities

Diagnosis[a]	Atrial fibrillation	Matched controls	*P*[c]
**Atherosclerotic heart disease (I25.1)**	253 (18.95)	195 (7.26)	<0.001
**Congestive heart failure (I50.0)**	50 (3.74)	4 (0.15)	<0.001
**Hyperthyreoidism (E05.0, E05.1, E05.2)**	< 5 (0.22)	< 5 (0.11)	0.660
**Diabetes mellitus type II, (E10, E11)**	120 (9.09)	185 (6.90)	0.023
**Alcohol abuse (F10.1, F10.2)**	23 (1.72)	31 (1.16)	0.184
**Chronic obstructive sleep apnea (G47.3)**	55 (4.12)	50 (1.86)	<0.001

[a]ICD10 codes in parentheses
[b]Presented as n (%)
[c]uncorrected *P*-values of $\chi 2$ tests

Table S3 – Group comparisons controlling for AF comorbidities

Measure	Atrial fibrillation[a]	Matched controls[a]	P_{uncorr}[b]	P_{FDR}[c]	*Cohen's d*
**Cognitive domains**					
Attention / executive dysfunction, z	-0.058 ± 0.851 (787)	0.039 ± 0.820 (1817)	0.023	0.047*	0.12
Information processing speed, z	-0.039 ± 0.720 (836)	0.031 ± 0.722 (1895)	0.116	0.154	0.10
Memory, z	-0.007 ± 0.570 (779)	0.012 ± 0.556 (1749)	0.710	0.710	0.03
Reasoning, z	-0.085 ± 0.837 (843)	0.049 ± 0.838 (1893)	0.001	0.004*	0.16
**Gray matter**					
Cortical thickness, mm	2.367 ± 0.087 (1293)	2.376 ± 0.088 (2594)	0.008	0.024*	0.10
Gyrification index	27.623 ± 0.501 (1293)	27.629 ± 0.517 (2594)	0.770	0.815	0.01
Sulcal depth, mm	9.134 ± 0.471 (1293)	9.135 ± 0.472 (2594)	0.959	0.959	0.00
Fractal dimension	2.613 ± 0.024 (1293)	2.614 ± 0.024 (2594)	0.352	0.512	0.04
Gray matter volume	0.458 ± 0.048 (1293)	0.461 ± 0.047 (2594)	0.083	0.150	0.06
Fractional anisotropy	0.144 ± 0.093 (1335)	0.144 ± 0.089 (2683)	0.370	0.512	-0.01
Mean diffusivity, 10^{-3} mm²/s	1.083 ± 0.080 (1335)	1.083 ± 0.076 (2683)	0.148	0.243	-0.01
Isotropic volume fraction	0.254 ± 0.041 (1335)	0.253 ± 0.039 (2683)	0.025	0.057	-0.03
Neurite density index	0.343 ± 0.024 (1335)	0.342 ± 0.021 (2683)	0.741	0.815	-0.02
Orientation dispersion	0.455 ± 0.018 (1335)	0.455 ± 0.017 (2683)	0.541	0.649	-0.00

**White matter**					
Fractional anisotropy (FA)	0.408 ± 0.016 (1335)	0.409 ± 0.018 (2683)	0.014	0.035*	0.05
Mean diffusivity (MD), 10^{-3} mm²/s	0.810 ± 0.033 (1335)	0.808 ± 0.33 (2683)	0.003	0.013*	-0.06
Isotropic volume fraction	0.086 ± 0.014 (1335)	0.085 ± 0.014 (2683)	<0.001	0.002**	-0.08
Neurite density index	0.554 ± 0.028 (1335)	0.554 ± 0.027 (2683)	0.458	0.589	0.00
Orientation dispersion	0.256 ± 0.011 (1335)	0.255 ± 0.012 (2683)	0.076	0.150	-0.04
White matter hyperintensity load, %	0.005 ± 0.005 (1215)	0.004 ± 0.004 (2645)	<0.001	<0.001***	-0.15
Peak width of skeletonized mean diffusivity, 10-3 mm²/s	0.257 ± 0.052 (1335)	0.251 ± 0.06 (2683)	<0.001	0.002**	-0.10

Abbreviations: z – z-score
[a]Presented as mean ± SD (N)
[b]Uncorrected P values of analyses of covariance, adjusted for age, sex, education, cardiovascular risk factors and AF comorbidities
[c]False discovery rate-corrected P values of analyses of covariance, adjusted for age, sex, education, cardio-vascular risk factors and AF comorbidities (*P <0.05, **P <0.01, ***P <0.001)

Table S4 – Group comparisons of individual cognitive tests

Clinical measure[a]	Atrial fibrillation	Matched controls	P_{uncorr}[b]	P_{FDR}[c]	*Cohen's d*
**Attention and executive function**					
Tower Rearranging Test	9.18 ± 3.24 (840)	9.55 ± 3.15 (1891)	0.008	**0.034***	0.12
Trail Making Test part B, seconds	664.67 ± 309.68 (804)	634.50 ± 295.77 (1855)	0.023	0.058	-0.10
**Processing speed**					
Reaction Time Test	6.71 ± 0.17 (1213)	6.72 ± 0.17 (2453)	0.139	0.231	0.04
Symbol Digit Substitution Test	16.68 ± 5.08 (848)	17.28 ± 5.04 (1915)	0.010	**0.034***	0.12
Trail Making Test part A, seconds	250.38 ± 91.47 (851)	248.11 ± 98.84 (1916)	0.904	0.904	-0.02
**Memory**					
Numeric Memory Test	6.48 ± 1.66 (883)	6.55 ± 1.61 (1977)	0.36	0.4	0.04
Paired Associate Learning Test	6.27 ± 2.68 (867)	6.44 ± 2.70 (1941)	0.221	0.316	0.06
Prospective Memory Test	1.08 ± 0.45 (1224)	1.06 ± 0.45 (2476)	0.256	0.319	-0.04
**Reasoning**					
Fluid Intelligence Test	6.34 ± 2.02 (1192)	6.49 ± 2.07 (2414)	0.034	0.068	0.07
Matrix Pattern Completion Test	7.40 ± 2.07 (848)	7.68 ± 2.07 (1911)	0.003	**0.027***	0.13

Abbreviations: z – z-score
[a]Presented as mean ± SD (N)
[b]Uncorrected P values of analyses of covariance, adjusted for age, sex and years of education
[c]False discovery rate-corrected P values of analyses of covariance, adjusted for age, sex, education and cardiovascular risk factors (*P <0.05, **P <0.01, ***P <0.001)

Figure S3 – Mediation analysis results

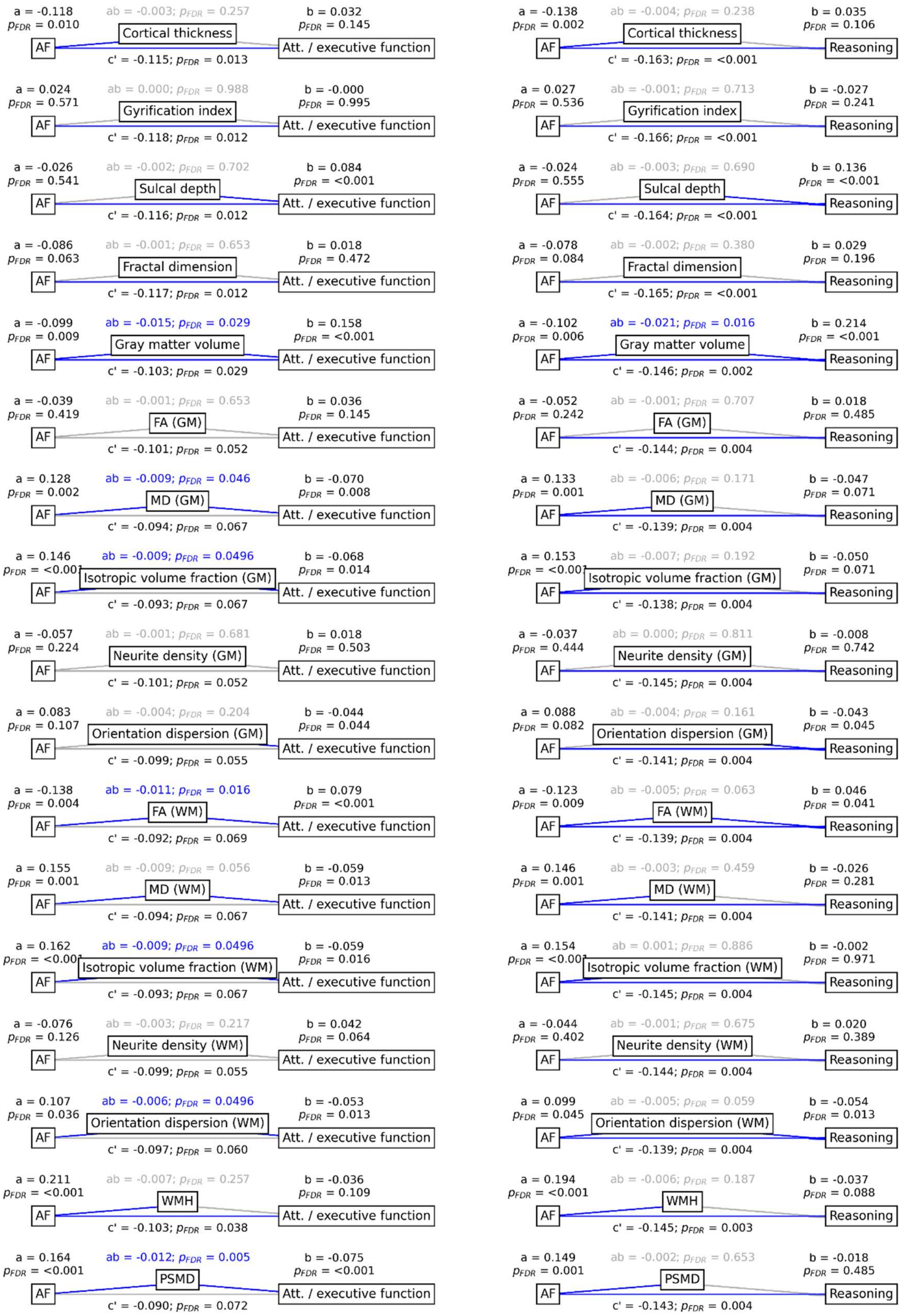

Mediation analysis results. Mediation effects of global imaging markers on the relationship between AF and attention/executive function as well as reasoning. Path plots display standardized effects and p-values: (a) AF to imaging marker, (b) imaging marker to cognitive score, (ab) indirect effect (c') direct effect and (c) total effect. Significant associations are in blue; non-significant in light gray. If a relationship is significantly mediated, i.e., the indirect effect ab was significant and the direct effect c' was reduced or non-significant compared to the total effect c, the text for ab is highlighted in blue. The left path plots show results regarding attention/executive function, while the right path plots depict those of reasoning.

Figure S4 – Regression analysis: cognition ~ cortical macrostructural imaging markers

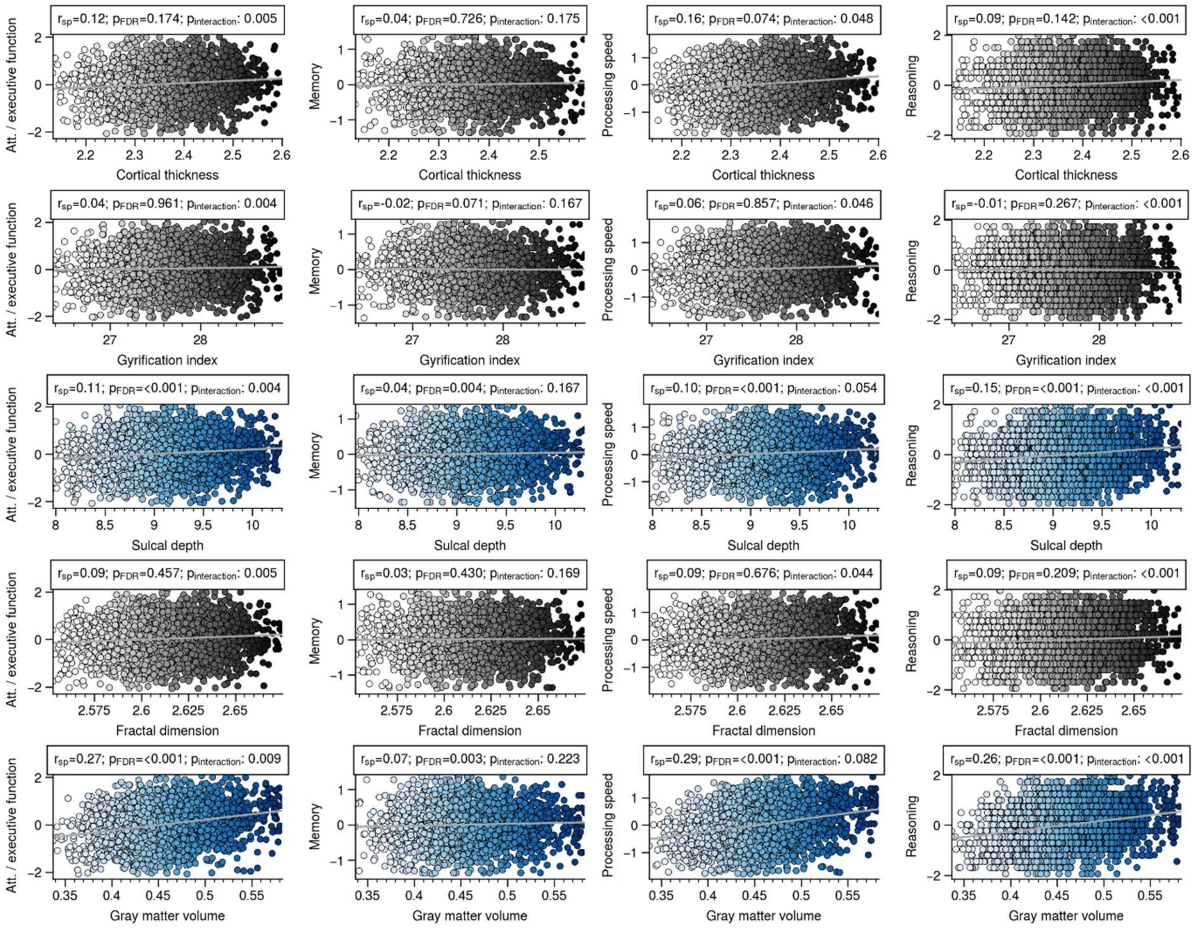

Linear regression analysis of the association between macrostructural cortical markers and cognitive outcomes. Analysis incorporated cognitive domain scores as the dependent variable, global imaging markers as the independent variable, and included age, sex, education and cardiovascular risk factors as covariates. The interaction effect of an atrial fibrillation diagnosis (global imaging marker * atrial fibrillation) was also tested. Significant associations between imaging and cognitive scores are denoted by blue dots. Non-significant associations are shown in gray. Abbreviations: p_{FDR} – false discovery rate corrected p-value, adjusted for age, sex, education, and cardiovascular risk; $p_{interaction}$ - p-value for the interaction term (imaging marker * atrial fibrillation) with false discovery rate correction; r_{sp} – Spearman correlation.

Figure S5 – Regression analysis: cognition ~ cortical microstructural imaging markers

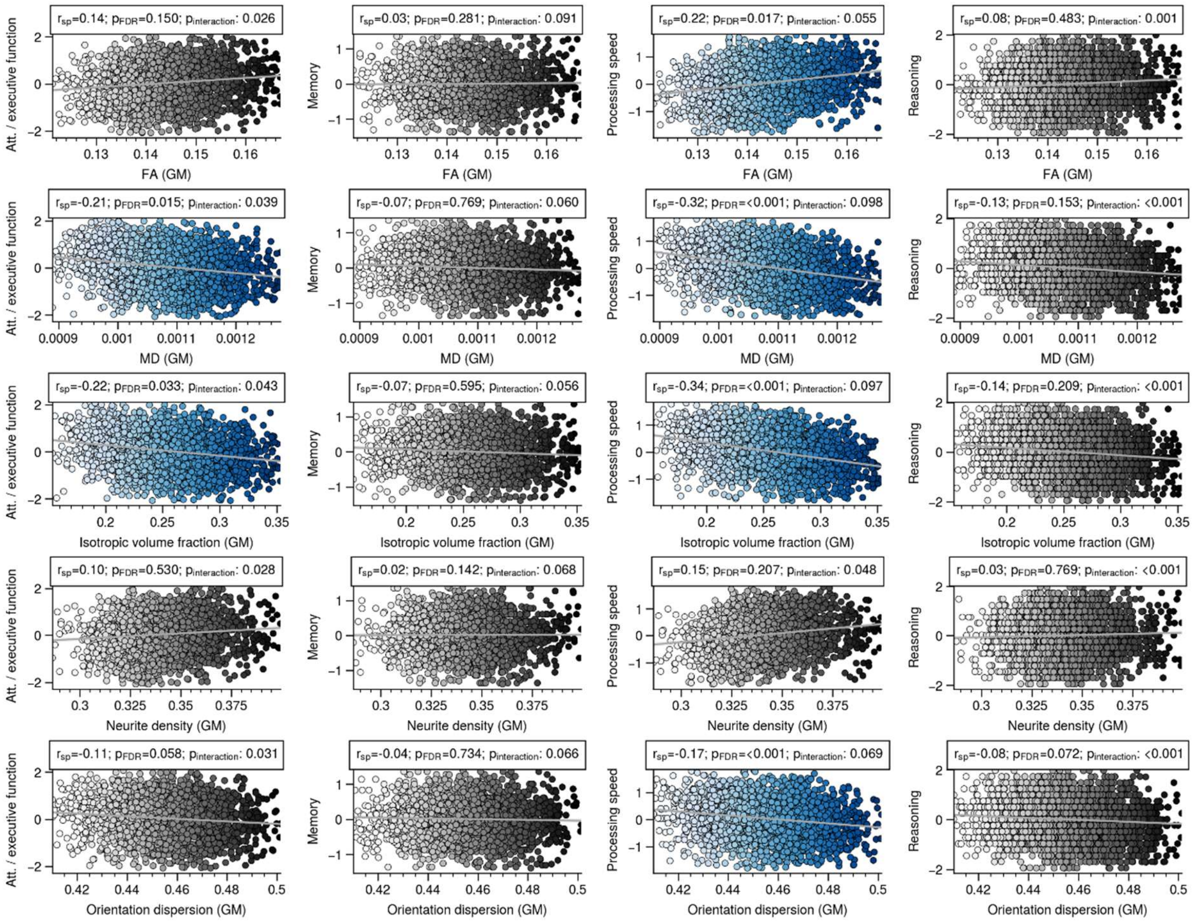

Linear regression analysis of the association between microstructural cortical markers and cognitive outcomes. Analysis incorporated cognitive domain scores as the dependent variable, global imaging markers as the independent variable, and included age, sex, education and cardiovascular risk factors as covariates. The interaction effect of an atrial fibrillation diagnosis (global imaging marker * atrial fibrillation) was also tested. Significant associations between imaging and cognitive scores are denoted by blue dots. Non-significant associations are shown in gray. Abbreviations: p_{FDR} – false discovery rate corrected p-value, adjusted for age, sex, education, and cardiovascular risk; $p_{interaction}$ - p-value for the interaction term (imaging marker * atrial fibrillation) with false discovery rate correction; r_{sp} – Spearman correlation.

Figure S6 – Regression analysis: cognition ~ white matter imaging markers

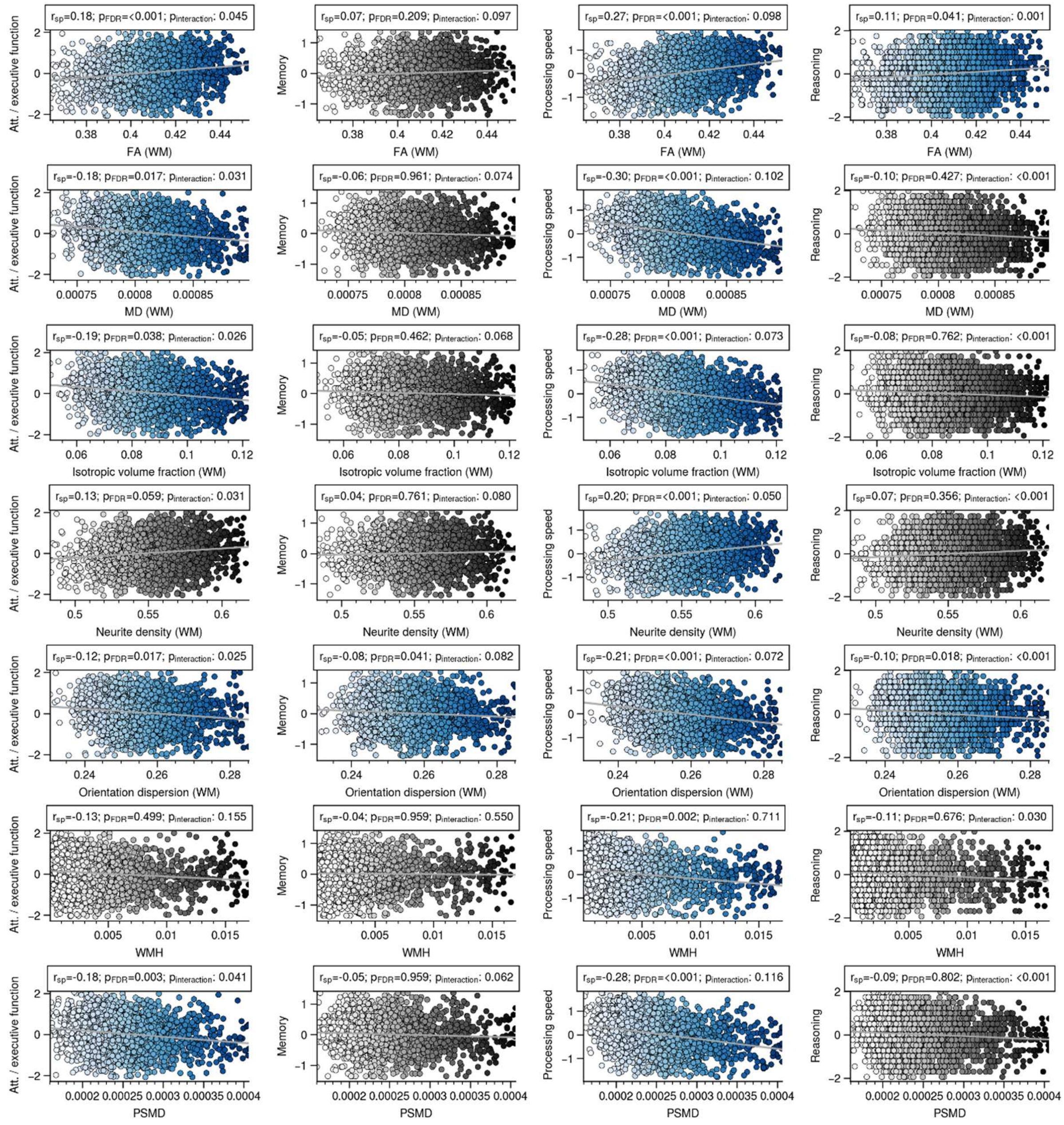

Linear regression analysis of the association between white matter markers and cognitive outcomes. Analysis incorporated cognitive domain scores as the dependent variable, global imaging markers as the independent variable, and included age, sex, education and cardiovascular risk factors as covariates. The interaction effect of an atrial fibrillation diagnosis (global imaging marker * atrial fibrillation) was also tested. Significant associations between imaging and cognitive scores are denoted by blue dots. Non-significant associations are shown in gray. Abbreviations: p_{FDR} – false discovery rate corrected p-value, adjusted for age, sex, education, and cardiovascular risk; $p_{interaction}$ - p-value for the interaction term (imaging marker * atrial fibrillation) with false discovery rate correction; r_{sp} – Spearman correlation.

Figure S7 – Cortical effect maps comparison

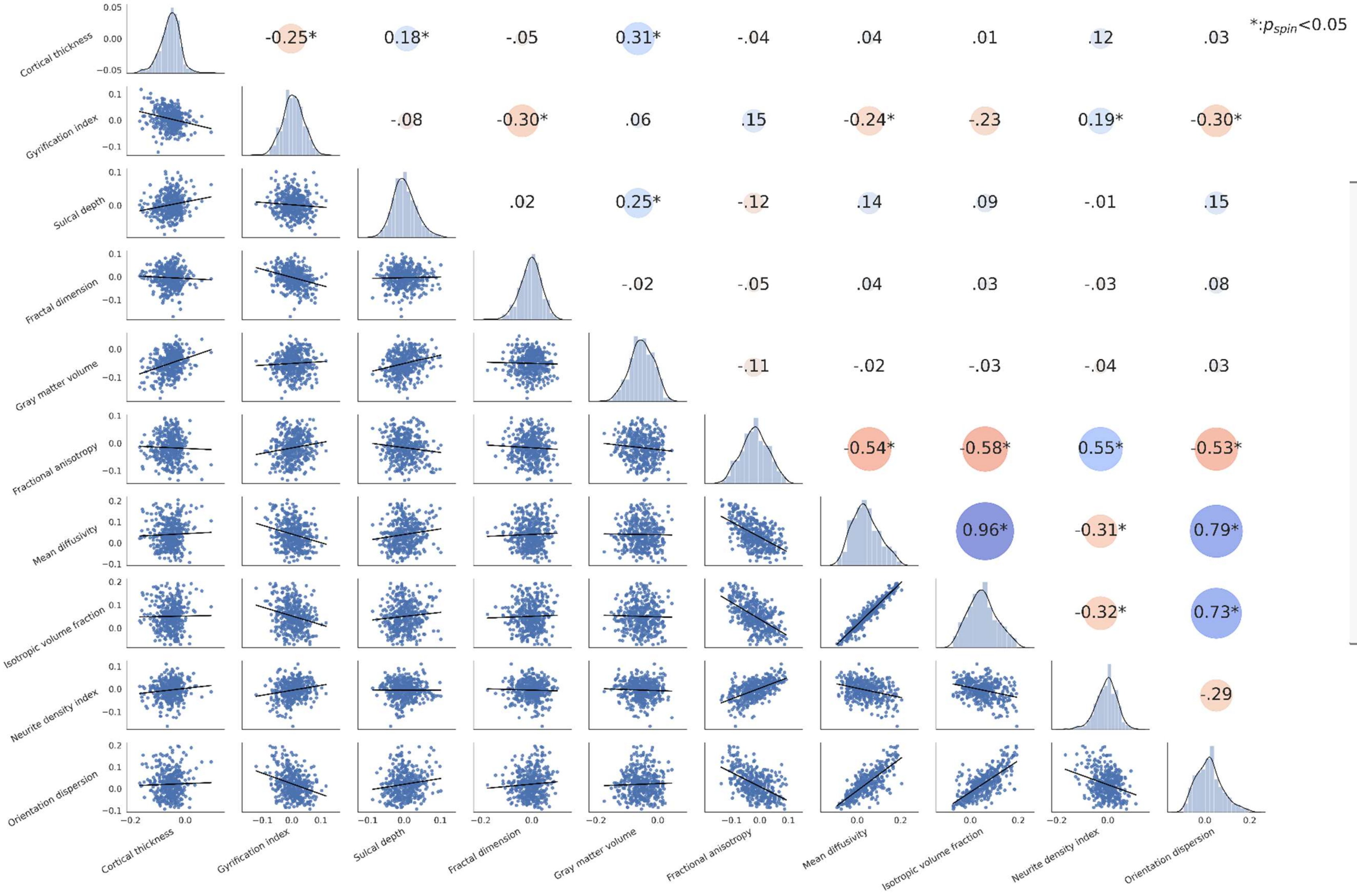

The correlation matrix displays spatial correlations of Schaefer400-parcellated Cohen's d maps resulting from the comparison of regional imaging markers between AF and controls. The lower triangle of the matrix displays scatter plots and the upper triangle the Pearson correlation coefficients of the corresponding correlations. The color and size of the dots on the upper triangle encode the effect directionality and effect size, respectively. The asterisks indicate statistical significance. To account for spatial smoothness, p-values were determined based on spin permutations (p_{spin}).[17] The matrix diagonal displays histograms.

Figure S8 - Group comparison of tract-level fractional anisotropy (FA)

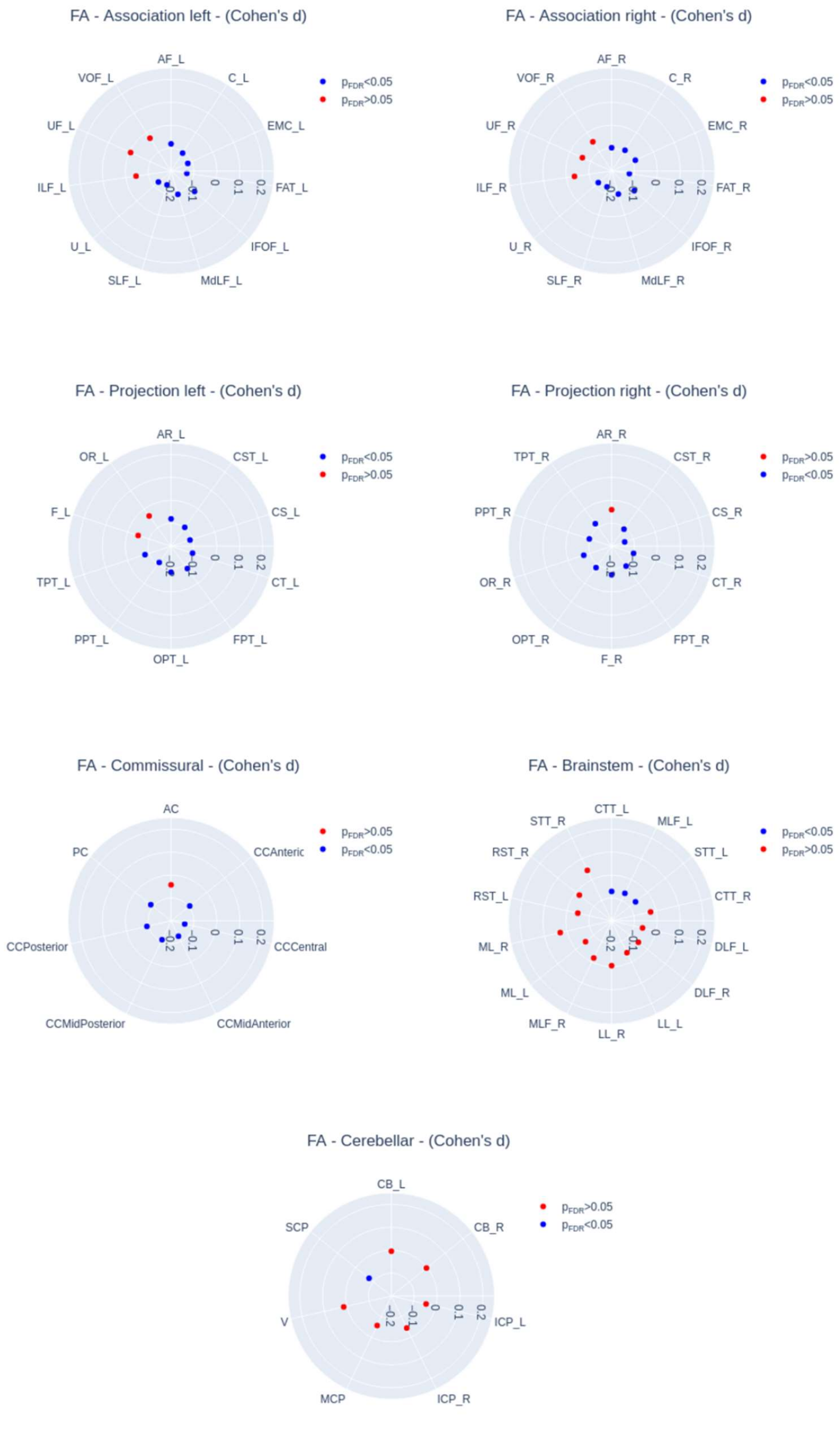

Radar plots represent the group differences for fractional anisotropy in each tract, with blue dots indicating significant differences and red dots marking non-significant differences.

Figure S9 - Group comparison of tract-level mean diffusivity (MD)

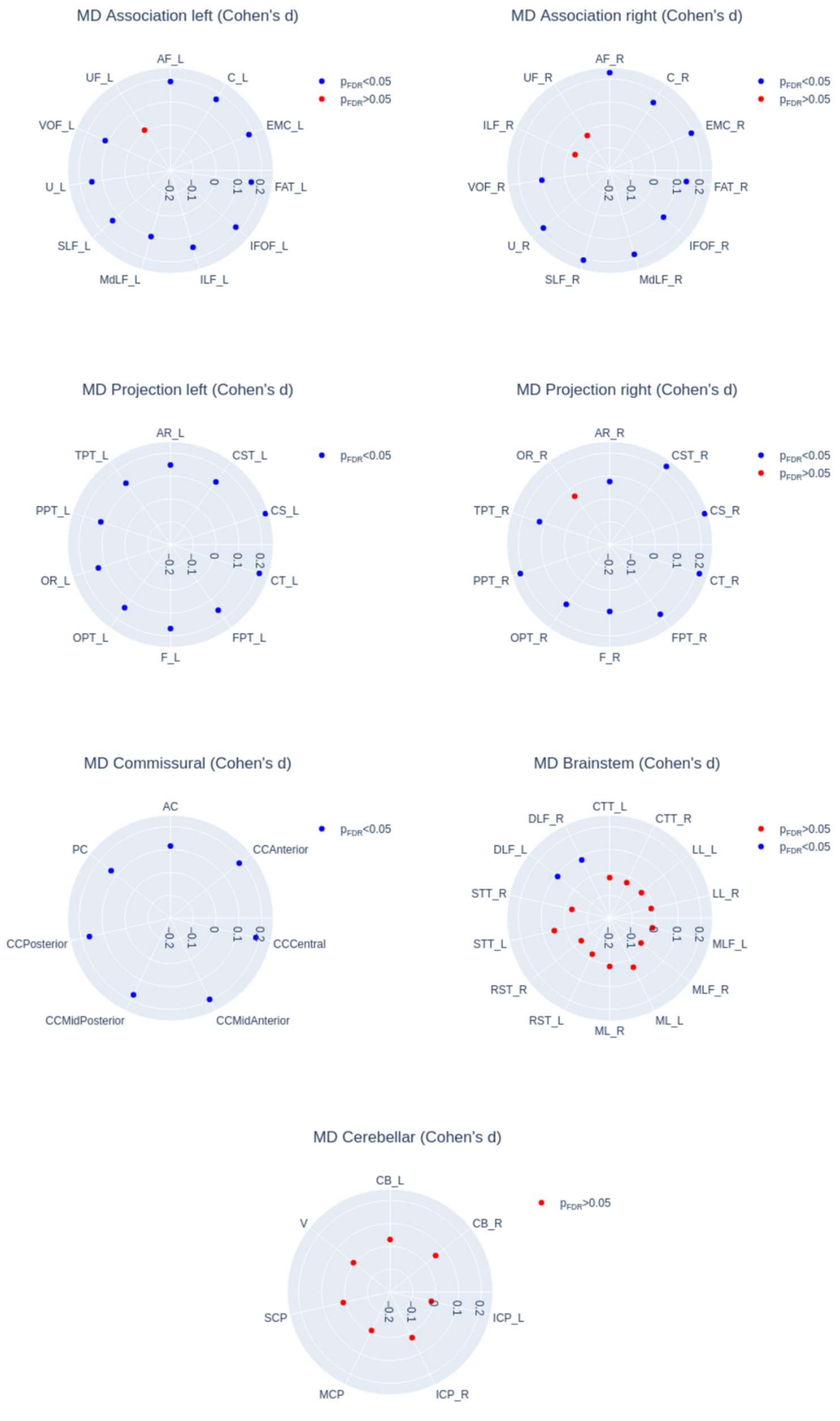

Radar plots represent the group differences for mean diffusivity in each tract, with blue dots indicating significant differences and red dots marking non-significant differences.

Figure S10 - Group comparison of tract-level neurite density index

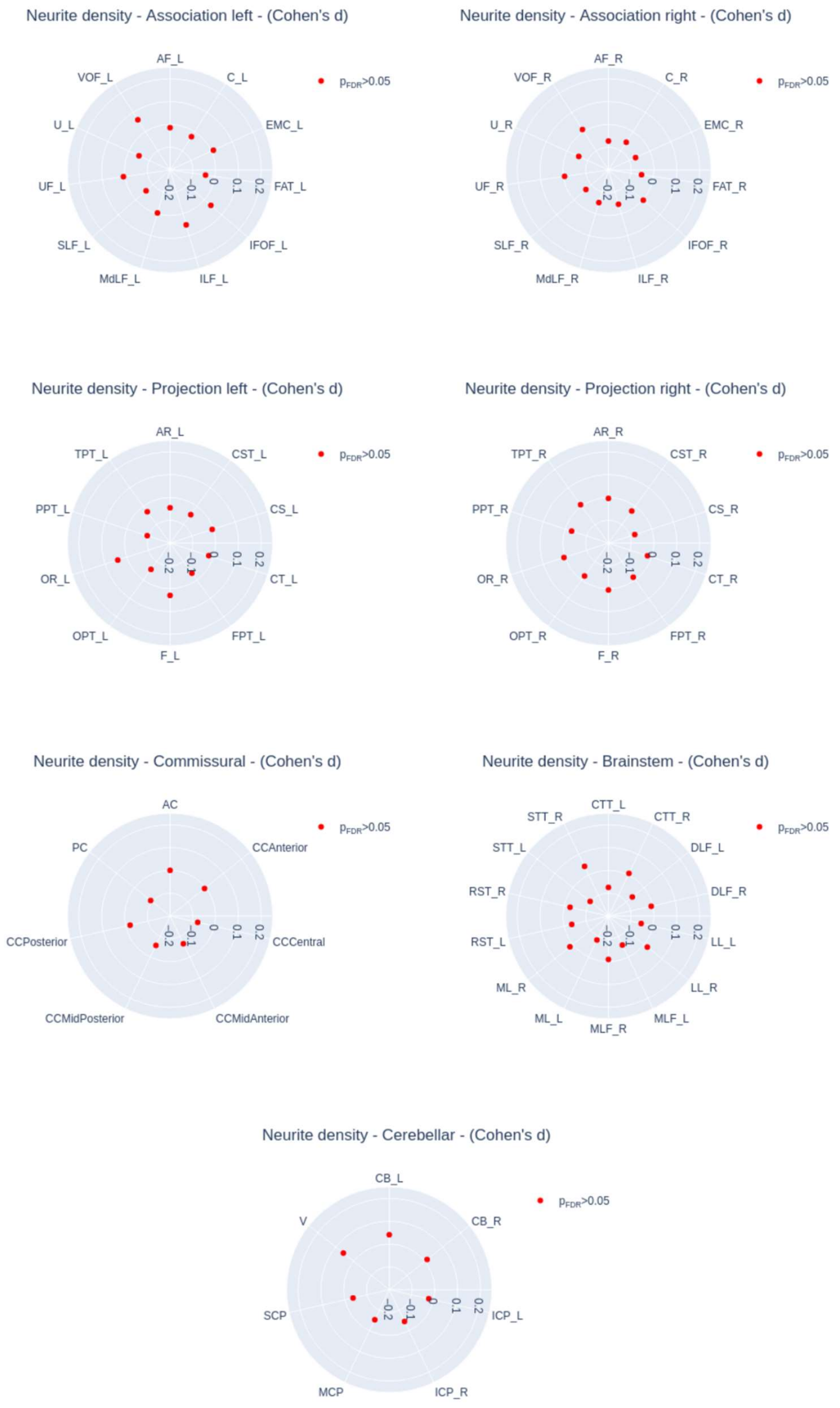

Radar plots represent the group differences of neurite density in each tract, with blue dots indicating significant differences and red dots marking non-significant differences.

Figure S11 - Group comparison of tract-level orientation dispersion

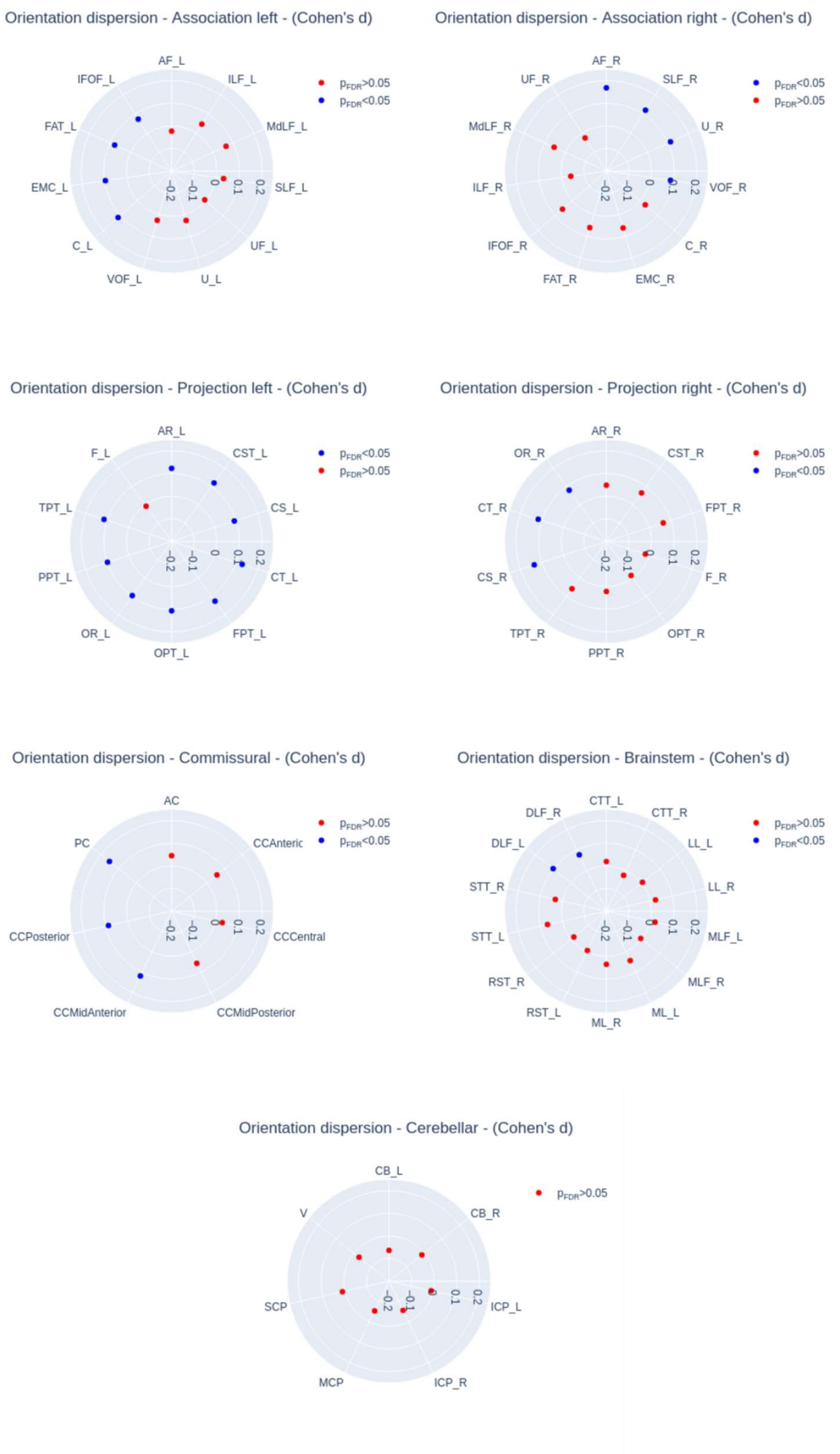

Radar plots represent the group differences for orientation dispersion in each tract, with blue dots indicating significant differences and red dots marking non-significant differences.

Discussion

Text S2 – Microstructural abnormalities map to specific cortical networks

Our study pinpointed AF-related variations in gray matter microstructure, with symmetrically impacted regions including the anterior cingulate, insula, medial prefrontal cortex, medial temporal lobe and precuneus. This observation becomes especially notable when contextualized with two pathomechanistic theories explaining its origin.

First, the pattern might be a manifestation of advancing AF-related cerebral pathophysiology. The anterior cingulate, medial temporal lobe and precuneus have a particular role in Alzheimer's and vascular dementia, directly influencing associations with cognitive symptoms.[18–21] It is plausible that pathology in these areas might accelerate cognitive decline and the onset of dementia in individuals with AF. As there is an observable disparity between cortical patterns of macro- and microstructural changes, we propose that these differences could represent varying local stages of tissue changes all tethered to a shared disease mechanism. An increase in extracellular free-water might act as a precursor to more pronounced neurodegenerative processes. Thus, the regions showing microstructural deviations in our current cross-sectional analysis might manifest macrostructural changes as the disease progresses. This interpretation finds support in studies assessing cortical thickness in diverse stages of vascular cognitive impairment. These studies demonstrate that thickness differences between mild and severe cases of vascular cognitive impairment coincide with the cortical regions highlighted by altered microstructure in our analysis which might be due to common disease mechanisms.[22,23] Further support comes from observations of microstructural indices being demonstrably highly sensitive to subtle disease alterations in vascular cognitive impairment and preceding morphological effects and lesion occurence.[24,25]

Second, on a more speculative note, the observed associative effects could also be attributed to neurodegenerative processes increasing the risk of AF. The regions we identified are all components of the central autonomic network (CAN), which plays a pivotal role in regulating

heart rate and contractility by transmitting autonomic signals.[26,27] Chronic ischemic and degenerative changes within the central autonomic network, might heighten the propensity for AF development. This perspective is supported by preclinical studies, which have demonstrated neurogenically triggered cardiac arrhythmias and changes in the left atrial myocardium post-stroke in rodent models.[28,29] Furthermore, in cases of AF detected after stroke (AFDAS), stroke lesions predominantly appear within the central autonomic network, pointing towards a potential neurogenic origin.[26] Notably, lesions in the right anterior insula correlate with post-stroke acute myocardial injury, underscoring the region's significance in brain-heart interactions.[30] In fact, the anterior insula is the only area showing significant group differences of neurite density in our analysis, implying greater intracellular volume loss possibly due to cellular and axonal damage. Longitudinal neuroimaging studies are needed to further substantiate these hypotheses in AF.

References

1. Gaser C, Dahnke R, Thompson PM, Kurth F, Luders E, Initiative ADN. CAT – A Computational Anatomy Toolbox for the Analysis of Structural MRI Data. Published online June 13, 2022:2022.06.11.495736. doi:10.1101/2022.06.11.495736

2. Dahnke R, Yotter RA, Gaser C. Cortical thickness and central surface estimation. *NeuroImage*. 2013;65:336-348. doi:10.1016/j.neuroimage.2012.09.050

3. Luders E, Thompson PM, Narr KL, Toga AW, Jancke L, Gaser C. A curvature-based approach to estimate local gyrification on the cortical surface. *Neuroimage*. 2006;29(4):1224-1230. doi:10.1016/j.neuroimage.2005.08.049

4. Van Essen DC. A Population-Average, Landmark- and Surface-based (PALS) atlas of human cerebral cortex. *NeuroImage*. 2005;28(3):635-662. doi:10.1016/j.neuroimage.2005.06.058

5. Yotter RA, Nenadic I, Ziegler G, Thompson PM, Gaser C. Local cortical surface complexity maps from spherical harmonic reconstructions. *NeuroImage*. 2011;56(3):961-973. doi:10.1016/j.neuroimage.2011.02.007

6. Ashburner J, Friston KJ. Diffeomorphic registration using geodesic shooting and Gauss-Newton optimisation. *Neuroimage*. 2011;55(3):954-967. doi:10.1016/j.neuroimage.2010.12.049

7. Kurth F, Luders E, Gaser C. Voxel-Based Morphometry. In: Toga AW, ed. *Brain Mapping*. Academic Press; 2015:345-349. doi:10.1016/B978-0-12-397025-1.00304-3

8. Andersson JLR, Sotiropoulos SN. An integrated approach to correction for off-resonance effects and subject movement in diffusion MR imaging. *Neuroimage*. 2016;125:1063-1078. doi:10.1016/j.neuroimage.2015.10.019

9. Basser PJ, Pierpaoli C. Microstructural and physiological features of tissues elucidated by quantitative-diffusion-tensor MRI. *J Magn Reson B*. 1996;111(3):209-219. doi:10.1006/jmrb.1996.0086

10. Basser PJ, Mattiello J, LeBihan D. MR diffusion tensor spectroscopy and imaging. *Biophys J*. 1994;66(1):259-267. doi:10.1016/S0006-3495(94)80775-1

11. Zhang H, Schneider T, Wheeler-Kingshott CA, Alexander DC. NODDI: Practical in vivo neurite orientation dispersion and density imaging of the human brain. *NeuroImage*. 2012;61(4):1000-1016. doi:10.1016/j.neuroimage.2012.03.072

12. Daducci A, Canales-Rodríguez EJ, Zhang H, Dyrby TB, Alexander DC, Thiran JP. Accelerated Microstructure Imaging via Convex Optimization (AMICO) from diffusion MRI data. *Neuroimage*. 2015;105:32-44. doi:10.1016/j.neuroimage.2014.10.026

13. Smith SM, Jenkinson M, Johansen-Berg H, et al. Tract-based spatial statistics: Voxelwise analysis of multi-subject diffusion data. *NeuroImage*. 2006;31(4):1487-1505. doi:10.1016/j.neuroimage.2006.02.024

14. Jenkinson M, Beckmann CF, Behrens TEJ, Woolrich MW, Smith SM. FSL. *Neuroimage*. 2012;62(2):782-790. doi:10.1016/j.neuroimage.2011.09.015

15. Baykara E, Gesierich B, Adam R, et al. A Novel Imaging Marker for Small Vessel Disease Based on Skeletonization of White Matter Tracts and Diffusion Histograms. *Annals of Neurology*. 2016;80(4):581-592. doi:10.1002/ana.24758

16. Griffanti L, Zamboni G, Khan A, et al. BIANCA (Brain Intensity AbNormality Classification Algorithm): A new tool for automated segmentation of white matter hyperintensities. *Neuroimage*. 2016;141:191-205. doi:10.1016/j.neuroimage.2016.07.018

17. Alexander-Bloch AF, Shou H, Liu S, et al. On testing for spatial correspondence between maps of human brain structure and function. *NeuroImage*. 2018;178:540-551. doi:10.1016/j.neuroimage.2018.05.070

18. Chen Y, Dang M, Zhang Z. Brain mechanisms underlying neuropsychiatric symptoms in Alzheimer’s disease: a systematic review of symptom-general and –specific lesion patterns. *Molecular Neurodegeneration*. 2021;16(1):38. doi:10.1186/s13024-021-00456-1

19. Flores R de, Das SR, Xie L, et al. Medial Temporal Lobe Networks in Alzheimer’s Disease: Structural and Molecular Vulnerabilities. *J Neurosci*. 2022;42(10):2131-2141. doi:10.1523/JNEUROSCI.0949-21.2021

20. Green ZD, Vidoni ED, Swerdlow RH, Burns JM, Morris JK, Honea RA. Increased functional connectivity of the precuneus in individuals with a family history of Alzheimer’s disease. *Alzheimer’s & Dementia*. 2021;17(S1):e054286. doi:10.1002/alz.054286

21. Jobson DD, Hase Y, Clarkson AN, Kalaria RN. The role of the medial prefrontal cortex in cognition, ageing and dementia. *Brain Communications*. 2021;3(3):fcab125. doi:10.1093/braincomms/fcab125

22. Thong JYJ, Du J, Ratnarajah N, et al. Abnormalities of cortical thickness, subcortical shapes, and white matter integrity in subcortical vascular cognitive impairment. *Human Brain Mapping*. 2014;35(5):2320-2332. doi:10.1002/hbm.22330

23. Seo SW, Ahn J, Yoon U, et al. Cortical Thinning in Vascular Mild Cognitive Impairment and Vascular Dementia of Subcortical Type. *Journal of Neuroimaging*. 2010;20(1):37-45. doi:10.1111/j.1552-6569.2008.00293.x

24. Mayer C, Frey BM, Schlemm E, et al. Linking cortical atrophy to white matter hyperintensities of presumed vascular origin: *Journal of Cerebral Blood Flow & Metabolism*. Published online December 1, 2020. doi:10.1177/0271678X20974170

25. Maillard P, Fletcher E, Lockhart SN, et al. White Matter Hyperintensities and their Penumbra Lie Along a Continuum of Injury In The Aging Brain. *Stroke*. 2014;45(6):1721-1726. doi:10.1161/STROKEAHA.113.004084

26. Sposato LA, Hilz MJ, Aspberg S, et al. Post-Stroke Cardiovascular Complications and Neurogenic Cardiac Injury: JACC State-of-the-Art Review. *Journal of the American College of Cardiology*. 2020;76(23):2768-2785. doi:10.1016/j.jacc.2020.10.009

27. Reisert M, Weiller C, Hosp JA. Displaying the autonomic processing network in humans – a global tractography approach. *NeuroImage*. 2021;231:117852. doi:10.1016/j.neuroimage.2021.117852

28. Balint B, Jaremek V, Thorburn V, Whitehead SN, Sposato LA. Left atrial microvascular endothelial dysfunction, myocardial inflammation and fibrosis after selective insular cortex ischemic stroke. *International Journal of Cardiology*. 2019;292:148-155. doi:10.1016/j.ijcard.2019.06.004

29. Oppenheimer S. Cerebrogenic cardiac arrhythmias: *Clin Auton Res*. 2006;16(1):6-11. doi:10.1007/s10286-006-0276-0

30. Krause T, Werner K, Fiebach JB, et al. Stroke in right dorsal anterior insular cortex Is related to myocardial injury. *Ann Neurol*. 2017;81(4):502-511. doi:10.1002/ana.24906
